# Biases in health expectancies due to educational differences in survey participation of older Europeans: It’s worth weighting for

**DOI:** 10.1007/s10198-019-01152-0

**Published:** 2020-01-27

**Authors:** Sonja Spitzer

**Affiliations:** Wittgenstein Centre for Demography and Global Human Capital (Univ. Vienna, IIASA, VID/ÖAW), International Institute for Applied Systems Analysis (IIASA), Schloßplatz 1, 2361 Laxenburg, Austria

**Keywords:** Activity limitations, Education and inequality, Health expectancies, Survey participation, Iterative proportional fitting (IPF), Survey of Health, Ageing and Retirement in Europe (SHARE), C83, I18, I31, J14

## Abstract

Health expectancies are widely used by policymakers and scholars to analyse the number of years a person can expect to live in good health. Their calculation requires life tables in combination with prevalence rates of good or bad health from survey data. The structure of typical survey data, however, rarely resembles the education distribution in the general population. Specifically, low-educated individuals are frequently underrepresented in surveys, which is crucial given the strong positive correlation between educational attainment and good health. This is the first study to evaluate if and how health expectancies for 13 European countries are biased by educational differences in survey participation. To this end, calibrated weights that consider the education structure in the 2011 censuses are applied to measures of activity limitation in the Survey of Health, Ageing and Retirement in Europe. The results show that health expectancies at age 50 are substantially biased by an average of 0.3 years when the education distribution in the general population is ignored. For most countries, health expectancies are overestimated; yet remarkably, the measure underestimates health for many Central and Eastern European countries by up to 0.9 years. These findings highlight the need to adjust for distortion in health expectancies, especially when the measure serves as a base for health-related policy targets or policy changes.

## Introduction

Life expectancy continues to increase in Europe. We live longer, but do we live healthier? Answering this question is of utmost importance in the presence of demographic change. How long and how healthy we live is necessary information for public and private healthcare providers to plan health coverage and care services. Furthermore, policymakers are interested in the employability of older generations when adapting pension systems, in particular, when adjusting the retirement age. Whether we spend our additional life years in good or bad health is frequently analysed via health expectancy (HEX), an indicator that captures the number of years a person can expect to live in good health. This concept was developed half a century ago [1, 2] and has garnered increasing attention from both scholars and policymakers. For example, the European Commission aims to add 2 years of healthy life for the average European between 2010/2011 and 2020 to improve the sustainability of the European social and healthcare systems [3, 4]. Furthermore, many European governments use HEX to set health-related targets and make policy changes based on this measure [5].

HEX usually combines information on mortality with prevalence rates of good or bad health from survey data; therefore, it captures both the quantity and quality of additional life years. A key problem with this approach, however, is that survey participation is often selective and differs by individual characteristics such as gender, age and socio-economic status. A common deviation is that highly educated individuals are more likely to participate in surveys than low-educated individuals, leading to an overrepresentation of the highly educated among the respondents [6–8]. This mismatch is crucial given the strong positive correlation between educational attainment and good health [9–12]. Overrepresenting healthy, well-educated individuals in surveys makes countries appear to have healthier populations than is actually the case.

The aim of this study is to explore if and how HEX differs when the education structure in the general population is considered. For this purpose, prevalence rates of bad health from the Survey of Health, Ageing and Retirement in Europe (SHARE) for 13 European countries are adjusted with calibrated weights based on auxiliary information from censuses. Although there has been vast research on HEX, to the best of my knowledge, no previous work has addressed whether biases in the education composition distort the measure. Given the widespread use of HEX among scholars and health authorities, knowing the reliability of the indicator in the context of flawed survey data is pivotal. Moreover, this study contributes to the literature by illustrating how bias can be adjusted for when auxiliary information on the true population structure is available.

## Background

### Educational attainment affects health

The positive correlation between educational attainment and good health is well established [9]. For example, the average life expectancy at birth of well-educated Europeans is 7 years higher than that of low-educated individuals [13]. Furthermore, low-educated persons report higher activity limitations [14] and higher levels of bodily pain [12]. This can be partially explained with economic rationales, such as the positive link between education and income or correlations between education and occupational choice [11]. Additionally, differences in health behaviour are potential drivers of the education gradient in health. On one hand, low-educated persons are more likely to smoke, drink heavily, and be obese than highly educated persons. On the other hand, they are less likely to use preventive care, drive safely, and live in safe houses [15]. While the positive relationship between socio-economic advantages and health is found throughout Europe, the magnitude of that correlation varies by gender and country. First, the education gradient is larger for men than for women in life expectancy [16] as well as in HEX [17]. Second, in Central and Eastern European (CEE) countries, highly educated individuals are much healthier than low-educated individuals; whereas the difference is small in, for example, Denmark [18]. While most social health inequalities among older Europeans are driven by current socio-economic conditions, childhood circumstances also add to the health differences between socio-economic groups [19].

### Educational attainment affects survey participation

Educational attainment is associated not only with health but also with survey participation. Low-educated persons are frequently underrepresented in health surveys, for example, in Belgium [7, 20], Denmark [21], and Finland, where the gap in survey participation between low- and well-educated individuals has substantially widened over time [6]. This violation of the “missing at random” assumption can be attributed to coverage errors, sampling errors, and non-response errors [22]. Coverage errors stem from the mismatch between the survey’s target population and its sampling frame, for example, when phone registers serve as sampling frames, although low-educated persons are less likely to own phones than the highly educated. Sampling errors denote the gap between sampling frame and the sample, which emerges because not all individuals in the sampling frame can be surveyed due to time and money constraints. To account for the unequal selection probabilities of sample units, surveys frequently provide sampling weights. Finally, non-response errors stem from differences between the invited sample and the actual respondents.

The strong association between non-response and low education [23] can be explained by three channels [22]. First, low-educated persons are harder to contact due to their socio-demographic and social–environmental attributes. For example, they might have unstable life paths and are consequently more likely to change their address. Second, participation in surveys is usually voluntary and low-educated persons are more likely to refuse to participate than the highly educated. Finally, low-educated individuals may be less likely to provide the requested survey data for reasons such as being too sick to participate or because they are less aware of certain domains such as their health or financial situation.

Education is not the only characteristic corresponding with lower response rates. Gender and age also impact survey participation, which is why these variables are commonly considered in survey weights. Furthermore, characteristics such as race [24] and relationship status [8] are associated with non-response. This study, however, only focuses on education-related biases. First and foremost, education is a common proxy for socio-economic status that is rather stable over lifetime with relatively low measurement error. Furthermore, the education gradient in response behaviour is well established. Finally, register or census data on the education structure in the general population are more readily available than auxiliary information on other socio-economic characteristics, making it more possible to compare the education distribution in the general population to that in the survey data.

### Educational differences in survey participation bias the prevalence of good and bad health

In summary, highly educated individuals are, on average, healthier than low-educated individuals and are more likely to participate in surveys. Thus, both the variable of interest (health) and the likelihood to participate in a survey are influenced by educational attainment. When inferences about the health of the general population are made based on unweighted prevalence rates from such flawed surveys, the general population appears healthier than what is true in reality. For example, Van Der Heyden et al. [20] found that the prevalence of people with diabetes and asthma in Belgium is underestimated when the actual education distribution in the general population is not considered. In the Netherlands, education-related non-response leads to negative biases in the prevalence of low self-assessed health, smoking, alcohol intake, and low physical activity [25].

### Prevalence of good or bad health is needed to calculate HEX

Prevalence rates of good or bad health are one of the main components needed when calculating HEX, which makes the education-related bias in survey data a major concern. Similar to life expectancy, HEX varies substantially among European countries and is particularly low in CEE countries [26]. Around 2010, HEX at birth was 70.1 years for Swedish men but only 52.6 years for Slovakian men. For women, HEX at birth ranged from 71.5 years in Malta to 52.7 years in Slovakia [27]. Overall, women live a larger proportion of their life disabled than men [28, 29].

While life expectancy has clearly increased throughout Europe, evidence on HEX is less conclusive. The outcome depends on the health dimension that is considered [30] as well as the survey utilised [31]. Analysing 25 European countries between 2005 and 2010, [30] show that years in poor general self-rated health at age 65 decreased by 0.5 (1.1) years for men (women). By contrast, years with chronic morbidity increased at the same time and years without activity limitations remained stable. Analysing the latter separately for different countries, Jagger [32] found that HEX increased in some countries but decreased in others. In addition to differences in health measures, surveys, sub-populations and the relationship between mortality and morbidity, the lack of a consistent time trend in HEX might be partly explained by the small number of observations in the surveys utilised. Analysing prevalence by country, gender, and age requires sufficient numbers of observations in each country–gender–age cell. This is often not the case, especially at older ages. Consequently, prevalence rates based on these small cells are often not reliable and have large confidence intervals: the small cell sizes make it difficult to separate the signal from the noise.

Regardless of the evidence on the inadequate representation of the low-educated persons in surveys, studies typically do not adjust for prevalence rates of HEX. One explanation for this might be that auxiliary information on the actual education distribution in the general population is not readily available. Register data are only accessible for some European countries and censuses are only conducted with long time intervals. Yet whenever available, auxiliary data on the actual education distribution in the general population can be utilised to calibrate weights so that they account for deviations between the true distribution and the survey distribution.

## Data

The following sections describe analyses of whether adjusting for the education structure in the general population changes the prevalence of bad health and consequently the HEX for European countries. The analyses rely on three different data sources. Auxiliary information that is expected to capture the actual education distribution in the general population is taken from Eurostat’s Census database, which provides Population and Housing Censuses for Europe. These census data are used to generate calibrated weights via iterative proportional fitting (IPF). In addition, life tables from Eurostat [33] along with prevalence of bad health from SHARE are taken to compute HEX with Sullivan’s method [2, 34]. Analyses and comparisons of HEX in Europe are frequently based on SHARE [26, 35, 36] as well as on the European Statistics on Income and Living Conditions (EU-SILC) and on the European Health Interview Survey (EHIS). This analysis utilises SHARE, because its sampling and weighting procedure is well documented, thus enabling an exact replication of the calibration approach employed [37, 38].

### The Survey of Health, Ageing and Retirement in Europe (SHARE)

Prevalence rates of bad health are extracted from the fourth wave of SHARE, which was mainly conducted in 2011, and consequently corresponds with the census data [39–42]. Although some interviews took place in 2010 and 2012, 94% of all observations stem from 2011. In total, 16 European countries participated in the fourth wave; however, 3 of these countries do not provide reliable census data via Eurostat for the requested year (see “Eurostat data for post-stratification weights and life tables”). Therefore, the analysis is restricted to 13 countries including Austria, Belgium, Czechia, Denmark, Estonia, France, Germany, Hungary, Italy, Poland, Portugal, Slovenia, and Spain.

The target population of SHARE consists of all non-institutionalised individuals aged 50 and older who regularly live in the respective survey country and speak its language(s). Spouses of target individuals are included in the data regardless of their age; however, for this study, all individuals younger than 50 years old are excluded [42–44]. The remaining number of respondents lies between 1615 in Germany and 6754 in Estonia. Some countries only provide small numbers of observations per gender–age–education cell, especially at higher ages. Respondent numbers for Germany, Poland, and Portugal are particularly small: all three countries provide less than 2000 observations. Germany and Poland also have small respondent numbers at ages 50–54, because their panel was not refreshed since Wave 2 in 2007. Details on the number of respondents for each country are summarised in Appendix 1.1. All numbers for SHARE refer to the final set of respondents used for the calculations in this paper.

The survey is based on probability samples with close to full target population coverage for all countries, yet details regarding the sample design, in particular the sampling frame, vary by country (for an overview, see [38, 43, 44]). Respondents were surveyed in their homes by interviewers using computer-assisted personal interviews. For details on response rates, consult [44].

For the calibration of weights, information on the proportions of respondents by country, gender, age, and educational attainment is required. Educational attainment is split into three groups in accordance with the International Standard Classification of Education [45]. The “low-educated” group includes individuals whose educational attainment is lower secondary education and less. The “medium-educated” group includes individuals with upper secondary or post-secondary non-tertiary education. The “high-educated” group includes all individuals with higher than post-secondary non-tertiary education. A fourth category was added to capture all individuals with missing values in their education variable (2.2%). The education categories are directly comparable to the categories in the census data. By construction, country information has no missing values in SHARE. The gender variable also has no missing values. Age information is available for all observations save four individuals in Czechia, who are subsequently excluded. To calculate proportions in SHARE for IPF, age is grouped into 10-year age groups with 90 + serving as an open-ended category. Details regarding the survey proportions by country, gender, age, and education are presented in Appendix 1.1.

HEX in Europe is most commonly calculated based on the Global Activity Limitation Indicator (GALI) [5, 27, 46, 47], making the health measure the obvious choice for this analysis. Moreover, evaluations show that GALI similarly measures function and disability across European countries [48, 49], allowing cross-country comparisons. In particular, GALI is based on the reply to the following survey question: “For the past 6 months at least, to what extent have you been limited because of a health problem in activities people usually do?” The question is answered by each survey participant based on three categories: “severely limited”, “limited but not severely”, and “not limited”. For the purpose of this study, GALI is dichotomised into a binary variable with (1) “severely limited” and (0) “not severely limited”. Prevalence of bad health *π* is calculated by country, gender, and 5-year age group; 85 years of age serves as an open-ended category. In the final set of respondents, GALI has missing values for only 0.58% of the survey participants. Because there is no evidence of an education-related pattern in item non-response concerning GALI, this study only focuses on unit non-response.

GALI is a self-assessed health measure, and as such, is likely biased depending on the respondent’s individual characteristics [50–53] and cultural background [54–57]. Low-educated survey respondents are particularly prone to misreporting their health. Some evidence suggests that low-educated individuals have the tendency to overestimate their physical health; whereas, highly educated persons tend to underestimate their physical health [57]. If that is the case, the bias in HEX that is associated with underrepresentation of low education could appear smaller than it actually is, because low-educated individuals are overstating their physical abilities. Furthermore, self-assessed measures are often upward biased at older ages [57, 58], presumably due to peer effects [59]. Thus, as a robustness analysis, the prevalence of bad health is also estimated based on grip strength, a tested measure that is expected to be less biased by systematic misreporting. Despite GALI and grip strength measuring different health domains, additional calculations based on grip strength are expected to reveal if self-reported and tested health measures are equally biased by educational differences in survey participation.

Grip strength is primarily used to measure sarcopenia, the age-related decrease in muscle mass [60]. Furthermore, it is a strong predictor of mortality [61], mobility, and cognition [62]. While GALI only captures activity limitations, grip strength is often considered a proxy for overall health. In SHARE, grip strength is ascertained twice per hand for each participant via a handheld Smedley dynamometer (for details, see Ref. [63]). In accordance with the literature, the maximum of these four measurements is used for robustness analysis [61, 63, 64]. Grip strength is measured in kilograms, yet the calculation of HEX requires a binary outcome variable. Consequently, thresholds have to be applied, dividing the participants into groups of impaired and unimpaired. The European Working Group on Sarcopenia in Older People (EWGSOP) suggests cut-off values < 20 kg for women and < 30 kg for men to determine the onset of sarcopenia [60]. More recent evidence, however, suggests that such pragmatic thresholds do not fully capture critically weak hand grip [61]. Moreover, grip strength varies by factors such as body height and country of residence [63], implying that thresholds should be adapted accordingly. Because the purpose of this study is not to analyse grip strength as such, the pragmatic approach suggested by EWGSOP is deemed satisfactory. If the thresholds are indeed inaccurate, they would affect both the adjusted and unadjusted prevalence rates and, therefore, would not distort the results.

### Eurostat data for post-stratification weights and life tables

The calibration of weights requires auxiliary information on the actual population structure. To this end, it is assumed that the auxiliary information captures the true structure in the population with respect to certain characteristics such as gender, age, and education. For this study, the European Population and Housing Censuses are utilised as auxiliary data [65]. Along with the National Statistical Institutes, Eurostat combined national censuses from 2011 for 32 European countries and structured them in a comparable manner. Sixteen of these countries overlap with the countries from SHARE Wave 4. Because the Netherlands, Sweden, and Switzerland show irregularities in the census data provided by Eurostat, these countries are not included in the current analysis, leaving a sample of 13 countries.

For each country, population totals by gender, age, and education for individuals over 50 years of age are extracted from the censuses. The totals are used as control totals when calibrating weights. Some countries have missing information on educational attainment, which is why four education categories are constructed. The education groups “low educated”, “medium educated”, and “high educated” are based on the same criterion as adopted in SHARE, which are described in “The Survey of Health, Ageing and Retirement in Europe (SHARE)”. In addition, an education category denoted “unknown education” is created. Regarding gender and age, missing values are negligible, which is why this analysis is only based on the known population, and census cells for unknown gender and age are excluded. The census does not differentiate between institutionalised and non-institutionalised persons, which is why it is assumed that both groups are comparable. For details regarding the population proportions by country, gender, age, and education based on the censuses, consult Appendix 1.1.

In addition to prevalence rates, the calculation of HEX with Sullivan’s method relies on life tables provided by Eurostat for 2011 [33]. They are prepared to resemble standard abridged period life tables by country, gender, and 5-year age group, with 85 + considered an open-ended category.

### Education distribution in SHARE versus that in the censuses

By comparing the education distribution of participants in SHARE with that in the respective censuses, three country groups can be differentiated: countries for which SHARE data fit the education distribution in the population, country data in which highly educated individuals are overrepresented and low-educated individuals are underrepresented, and remarkably, country data in which this trend is reversed. Tables comparing the distributions can be found in Appendix 1.1.

The only two SHARE datasets resembling the education distribution in the population are those for Italy and Spain. The fit for Italy is close to perfect (Table 9). Spain shows slight deviations in the younger age groups, but overall achieves concordance between SHARE and the census (Table 13). Both countries have little variation in education within age groups. For example, the vast majority of the 70 + population is low educated. This pattern might explain the good fit with respect to the education distribution. However, Portugal also has little variation in education within age groups, but the education distribution in SHARE varies strongly from that in the census (Table 11). Hence, non-complex education distributions do not guarantee concordance between the education structure in surveys and the general population.

For most countries, high-educated individuals are overrepresented and low-educated individuals are underrepresented in SHARE. This pattern is in line with the literature discussed in “Background”. The countries belonging to that category are Austria, Belgium, Denmark, Germany, Hungary, Portugal, and to a lesser extent France and Slovenia. The deviation is particularly strong in Denmark, where the proportions in SHARE differ from those in the census on average by 51% for men and 52% for women in the age group of 50–89 (Table 4).

Interestingly, three CEE countries show the opposite pattern. In Czechia, Estonia, and Poland, low-educated individuals are overrepresented in the survey. Deviations are minor for Estonia (Table 5) and Poland (Table 10). For Czechia, however, SHARE proportions deviate from the census by 95% for men and 38% for women on average (Table 3). While high-educated individuals are underrepresented in the Estonian and Polish data, medium-educated individuals are underrepresented in the Czech data. Overall, the findings presented in this subsection suggest a need for education-adjusted weights (EW) when making inferences based on survey data.

## Method

To determine if distortions in the education distribution of survey data affect HEX, SHARE sampling design weights are adjusted via IPF so that the education structure in SHARE would match the education structure in the respective census. Following that, two sets of prevalence rates of severe activity limitations are computed. The first set *π*^EW^ is calculated using EW; whereas the control set *π*^RW^ uses standard weights without adjustment. Finally, Sullivan’s method is applied to calculate HEX^EW^ with education-adjusted prevalence rates and HEX^RW^ with the unadjusted rates. Comparing the two sets of HEX reveals if and how the measure is biased by educational differences in survey participation.

### Generating calibrated weights via IPF

Frequently, the proportions of certain characteristics in survey data deviate from the proportions of the same characteristics in the general population. Assuming that the distribution in the general population is known, calibrated weights can be generated for each survey respondent to account for these discrepancies. Calibrated weights are usually based on sampling design weights, which compensate for unequal selection probabilities of sample units, and in the case of SHARE, are provided with the survey data. They are defined as the inverse of the probability of being included in the sample. These design weights account for the unequal selection of sample units, but not for unit non-response [43].

A common method for calibrating sampling design weights is IPF, also known as raking. For this approach, marginal totals for each variable on which the weights are calibrated are taken from an auxiliary source that is assumed to capture the true distribution in the general population. When applying IPF, sampling design weights are iteratively modified by a multiplicative factor until convergence is achieved and the marginal totals of the adjusted weights conform to the corresponding marginal totals from the auxiliary source [66, 67]. After the adjustment, groups that were formerly underrepresented have relatively larger weights; whereas groups that were formerly overrepresented have relatively smaller weights. Importantly, the original information provided by the sampling design weights is maintained, since the weights within a group increase proportionally. The empirical strategy of this study evolves around three different sets of calibrated weights, which are discussed in more detail below.

#### SHARE weights (SW)

SHARE provides its own set of calibrated weights to account for differences in response behaviour. However, their weights do not consider the education structure in the general population [38]. For the remainder of this paper, these weights are referred to as SHARE weights (SW). The SW are generated based on a calibration approach by Deville and Särndal [68], which is implemented using Stata’s sreweight command by [69]. Control totals for the SW stem from the Eurostat regional database. The weights are calculated separately for each country, considering NUTS 1 regions as well as eight gender–age groups, with cutoffs at 50–59 years, 60–69 years, 70–79 years, and an open-ended category of 80 + years. In some countries, finer partitions are made below age 59 [37, 38].

#### Replicated weights (RW)

In a first step, the SW are replicated; this second set of weights is referred to as replicated weights (RW). Using RW instead of SW ensures that differences between estimates with and without education-adjusted weights do not stem, for example, from methodological differences applied for SW and EW. The goal is for RW to be as close as possible to the SW. However, some amendments in the method are made, so that later, education could be added as an additional control total. First, control totals are used for each calibration variable separately, instead of cross-classification. For example, instead of using age–gender totals, separate totals for age and gender are applied. The rationale behind this modification in the method is that calibrated weights are generally less stable and less likely to converge when observations are thinly spread over the calibration cells [66]. Using separate totals increases the number of observations by calibration cell. As a second amendment, Stata’s survwgt rake algorithm by [67] is used to generate the RW because it appears more robust than the sreweight command [70]. Third, control totals for NUTS 1 regions are not considered in this study, again, to increase the weight’s stability. The control total was included for a robustness analysis but did not alter the results. Fourth, an additional age category of 80–89 years is included, making 90 + the open-ended category. Finally, the Eurostat regional database does not provide information by education, which is why the 2011 census is used for this paper instead. Although these five changes are made, prevalence rates calculated based on the SW are almost identical to those calculated based on the RW, which confirms the approach.

#### Education-adjusted weights (EW)

Following the replication of SW, the EW are calculated. They are identical to the RW, except that an additional control total for education is considered for the calibration. Hence, EW vary for each individual observation, depending on the individual’s sampling design weight, gender, age, and educational attainment. In addition, the 2.2% of individuals with missing values for education receive a calibrated weight, since both the prevalence rates by education and the control totals include a category for “unknown education”.

Weighted prevalence rates of bad health *π* are calculated based on RW (*π*^RW^) and EW (*π*^EW^). In particular, the prevalence rates for the main analysis are based on the binary GALI measures, and prevalence rates for the robustness analysis are based on dichotomised grip strength. The means are calculated separately by country, gender, and 5-year age group, which follows the most common approach to calculate HEX in Europe. Prevalence rates *π*^RW^ and *π*^EW^ based on GALI along with the confidence intervals are presented in Appendix 1.2.

### Calculating HEX with Sullivan’s method

HEX is computed by applying Sullivan’s method [2, 34]. According to the standard life table notation (e.g. [71]), let

*l*_*x*_ = number of survivors at exact age *x* (beginning of age interval *i*)

*L*_*i*_ = number of person-years lived in age interval *i*

*π*_i_ = prevalence of severe activity limitations in age interval *i*.

Then HEX at age *x* is calculated separately by country and gender as follows:

$${\text{HEX}}_{x} = \frac{1}{{l_{x} }} \mathop \sum \limits_{i = x}^{A} (1 - \pi_{i} ) \times L_{i} ,$$

where the 5-year age groups range from *i* = 0 to *A*. More specifically, prevalence rates *π*_*i*_ are used to divide person-years lived according to the Eurostat life tables into years with and without severe activity limitations. Following that, HEX is calculated by dividing the number of individuals surviving to a certain age *x* by the total years lived healthily from age *x* onwards. Two sets of HEX are calculated. HEX^EW^ is based on *π*^EW^, the prevalence of severe activity limitations in age interval *i* weighted with EW. HEX^RW^ is based on *π*^RW^, the prevalence of severe activity limitations in age interval *i* weighted with RW. The bias in HEX due to the misrepresentation of educational groups in the survey is computed as the difference between HEX^RW^ and HEX^EW^ and denoted as ∆HEX. Confidence intervals around HEX^RW^, HEX^EW^ and ∆HEX are approximated using the method suggested by [72].

An alternative to calculating HEX via Sullivan’s method is the multistate life table method, which is sometimes said to be more accurate [73, 74]; however, Mathers and Robine [75] report that differences between the two methods are small. Furthermore, Sullivan’s method is the most common approach to calculate HEX in Europe for both health authorities and scholars, which makes the results of this study comparable.

## Results

### Prevalence of bad health with and without adjusted weights

The differences between adjusted (*π*^EW^) and unadjusted (*π*^RW^) prevalence rates correspond to the deviation in education structure in SHARE from the census (see tables in Appendix 1.2). For Italy and Spain, *π*^RW^ and *π*^EW^ are rather similar. For all country datasets in which high-educated individuals are overrepresented and low-educated individuals are underrepresented, *π*^RW^ is smaller than *π*^EW^, indicating a downward bias in mean activity limitation. This finding is in line with the evidence that education and good health are positively correlated. The size of the bias depends on the deviation between SHARE data and the census. It is minor for countries such as France, where the deviation is small: *π*^RW^ at age 50 is 0.095 (0.097) for men (women) and *π*^EW^ at age 50 is 0.105 (0.107) for men (women). Yet the bias is severe for countries such as Denmark, where the deviation is large: *π*^RW^ at age 50 is 0.074 (0.076) for men (women) and *π*^EW^ at age 50 is 0.107 (0.110) for men (women).

For the three countries in which low-educated individuals are overrepresented, *π*^RW^ is larger than *π*^EW^, indicating an upward-bias in mean activity limitation. Consequently, these countries appear healthier once the education structure in the general population is considered. The countries concerned are Czechia, Estonia, and Poland. The shift is most pronounced for Czechia, which is in line with the finding that the Czech SHARE data are particularly distorted.

Confidence intervals of *π*^EW^ and *π*^RW^ are mostly overlapping due to the small numbers of observations in the age–gender–education cells. For example, the male age group 90 + in Germany only consists of five men, and that in Slovenia consists of four men only. In Austria, the male age group 90 + consisted of 20 men, of which 7 are low educated, 6 are medium educated, 6 are high educated, and 1 has unknown education. While the aggregated data show a clear positive link between educational attainment and good health, the direction of the relationship between education and health in these small gender–age cells is sometimes the opposite. For example, the seven low-educated men in the Austrian 90 + group reported on average better health than the six high-educated men. Due to the reversal, prevalence of bad health is slightly lower for that group, once EW are applied. Given the small number of observations in certain cells and the subsequently large confidence intervals, HEX as well as differences in HEX have to be interpreted cautiously, especially for Portugal and Germany, where confidence intervals are particularly large and no clear age gradient in severe activity limitations for men is visible.

Comparing prevalence rates based on grip strength measures with those based on GALI leads to similar findings as described above. Yet for most countries, the age gradient in bad health is steeper when measured via grip strength, so the prevalence of bad health at old age is usually higher. This finding could be explained with the evidence that participants rate their health relatively better at old age than at young age (see “The Survey of Health, Ageing and Retirement in Europe (SHARE)”). Most notably, Portuguese and German men show a clear age gradient in education when health is tested with grip strength, while no such age gradient is visible when health is measured with GALI.

### Bias in HEX

**Fig. 1:**
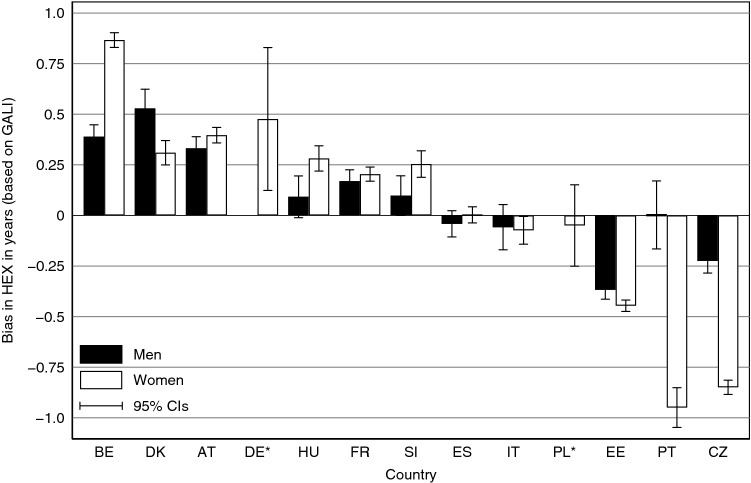
Bias in HEX based on GALI at age 50 in 2011. The bias is given in absolute years, i.e. ∆HEX is calculated as the difference between HEX^RW^ and HEX^EW^. *Results for German as well as Polish men are not shown, because small numbers of observations at ages 50–54 and subsequent large confidence intervals prevent a meaningful illustration and interpretation of the difference between HEX for those countries

Figure 1 shows how HEX at age 50 is biased because of educational differences in survey participation. The bias is given in absolute years and the countries are ranked based on the average bias in all age groups. Results for German as well as Polish men are not shown, because small numbers of observations at young ages and subsequent large confidence intervals prevent a meaningful illustration and interpretation of the difference in HEX for those countries at ages 50–54. In addition to Fig. 1, HEX^RW^ and HEX^EW^ are presented in Appendix 1.2 for all age groups, along with the respective bias in absolute years denoted as ∆HEX and the proportional bias denoted as ∆%. Confidence intervals for HEX^RW^, HEX^EW^ and ∆HEX are also provided in Appendix 1.2.

On average, HEX at age 50 is biased by 0.3 years, yet the deviation varies substantially between countries and genders. It is larger for women (0.4 years) than for men (0.2 years), presumably due to the higher life expectancy of women in general. For most parts, the bias resembles the deviations between SHARE and the census, and consequently, the deviation between *π*^RW^ and *π*^EW^. As a result, HEX^RW^ and HEX^EW^ are similar for Italy and Spain, since SHARE mimics the censuses in those countries. At age 50, ∆HEX for Spanish men (women) is only − 0.04 (0.00) years. For Italian men (women), the bias is only − 0.07 (− 0.06) years. Overall, the deviations are even smaller at older ages.

By contrast, HEX at age 50 is upward-biased in countries for which high-educated persons are overrepresented in the SHARE data. This is the case for Belgium, Denmark, Austria, Germany, Hungary, France, and Slovenia. Without EW, these countries appear to have a healthier population than is actually the case. At age 50, the upward bias is largest for women in Belgium, where HEX is overestimated by 0.87 years or 3.5%. The opposite is true for Estonia, Czech Republic, and Poland, where low-educated individuals are overrepresented in the SHARE data. Consequently, these countries appear unhealthier than they actually are. At age 50, the downward bias is largest for Czech women, whose HEX is 0.85 years or 3.2% lower when the education structure in the general population is ignored. Since the bias has different magnitudes, and more importantly, different directions, it affects the country ranking of HEX. For example, Danish men aged 50 appear to have relatively high HEX without the EW (rank 4 of 13) but drop to the lower middle field (rank 7 of 13) when adjustments are made.

∆HEX mostly decreases with age, since life expectancy decreases with age. The proportional bias ∆%, however, remains stable over all age groups or decreases only slightly for the most part. Overall, the country and gender differences described for age 50 also hold for older age groups. Due to uncertainty in the data, however, some age groups in some countries (e.g., male age group 90 + in Austria) do not show the expected sign for ∆HEX. As indicated in the previous sections, the results for Germany and Portugal have to be treated especially carefully due to the small cell sizes. HEX at age 50 for Portuguese men appears to be severely underestimated, although the data clearly show that high-educated men are overrepresented in the Portuguese SHARE data (Table 11).

**Fig. 2:**
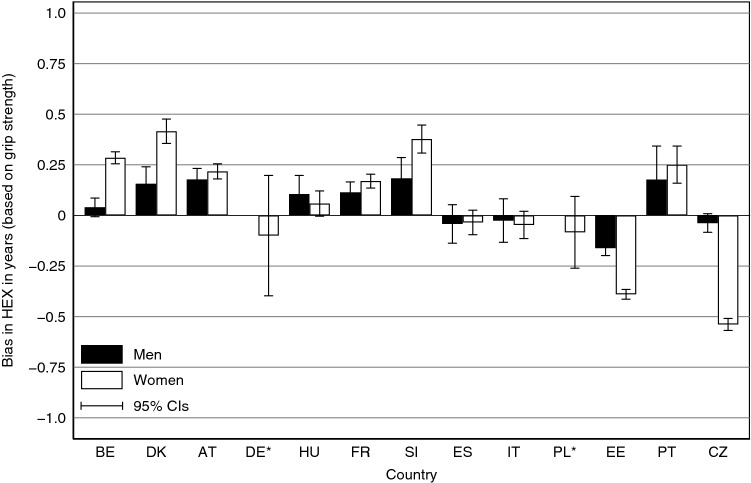
Bias in HEX based on grip strength at age 50 in 2011. The bias is given in absolute years, i.e. ∆HEX is calculated as the difference between HEX^RW^ and HEX^EW^. *Results for German as well as Polish men are not shown, because small numbers of observations at ages 50–54 and subsequent large confidence intervals prevent a meaningful illustration and interpretation of the difference between HEX for those countries

As a robustness analysis, HEX based on grip strength is also provided (Fig. 2). The overall bias appears smaller when the tested indicator is applied: average ∆HEX at age 50 is reduced to 0.17 years but is still larger for women (0.23 years) than for men (0.11 years). Even though the overall level of the bias is lower when grip strength is utilised, the general findings are confirmed. The bias is still negligible for Italy and Spain. The countries showing an upward bias based on GALI also show an upward bias based on grip strength; the same holds for all countries showing downward biases. Moreover, the inconsistencies in the Portuguese data disappear once grip strength is used. HEX at age 50 for both Portuguese men and women appears to be overestimated without the EW, just as expected when comparing the Portuguese SHARE data with the census. By contrast, the results for German women suggest an unexpected downward bias of HEX, albeit with a large confidence interval, which indicates once again that results based on small numbers of respondents must be handled with care.

## Discussion

This study is the first to evaluate if HEX in Europe is biased by educational differences in survey participation. The analysis showed that SHARE data for 11 of the 13 countries analysed did not resemble the education structure in the general population. In most countries, high-educated individuals were overrepresented, leading to an upward bias in HEX by up to 0.87 years, because of the positive correlation between educational attainment and good health. Contrary to what is suggested in the literature, most CEE countries analysed showed the opposite pattern that high-educated individuals were less likely to participate in surveys. As a consequence, HEX was underestimated by up to 0.85 years in those countries. These biases are crucially important, especially since HEX is frequently used by health authorities to assess population health and to make comparisons between countries. Future studies could fruitfully explore this issue further by exploring the non-response related bias in HEX for other surveys such as EHIS and EU-SILC. Investigating EU-SILC is considered particularly relevant since the data are used to monitor the European Commission’s aim to add 2 years of healthy life for the average European by 2020.

Related literature suggests that the biases are in fact larger and that the results ascertained in this study constitute a lower bound. First and foremost, this is because the low-educated individuals who participate in surveys are most likely healthier than the low-educated individuals who are not captured. Studies have shown that low-educated respondents have lower mortality [76], better self-reported health [77–79], and suffer less from psychosis [80] than low-educated non-respondents. Thus, being included in the survey is a collider that creates an artificial negative correlation between educational attainment and health. Importantly, this collider bias introduces an even larger bias for all countries in which high-educated persons are overrepresented. In addition, measurement errors in education might increase the biases. For example, [81] found that a substantial proportion of Danish SHARE participants exaggerated their level of education, especially when they were low educated. If unhealthy low-educated individuals exaggerate their level of education, they artificially narrow the health gap between low- and high-educated participants, adding to the bias. Finally, the survival bias might increase the bias in HEX if unhealthier low-educated persons have higher mortality and consequently do not appear in the survey.

An important finding of this study was that, in contrast to common results from the literature, low-educated individuals are not necessarily more likely to be underrepresented in surveys than the highly educated. The education structures in the Italian and Spanish SHARE are almost identical to those in the respective censuses. Consequently, HEX appears to be unbiased for these countries. Potentially, this is because educational attainment hardly varies within age groups in both nations, making it easier to survey the “correct” distribution. However, Portugal has similar education patterns across age but a still highly biased HEX. What could also explain the good fit for Italy and Spain is that the effect of education on health appears to be weaker than that for other countries. Both nations are among the countries with the highest life expectancy in Europe [33], even though their overall level of education is low compared to Western and Northern European countries [65]. Moreover, the education gradient in life expectancy is very pronounced in most of Europe; yet interestingly, Italy was the only country in the sample in which life expectancy at age 50 was slightly lower for the highly educated (34.6 years) than for the medium educated (35.2 years) [13]. Unfortunately, Eurostat does not provide life expectancy by education for Spain, thereby preventing a comparison. [16] found similar results for Italian women during the 1990s, although not for men. The evidence suggests that the association between education and health might be weaker in both countries than in other European countries. If the link between education and survey participation is weaker too, this would be an additional explanation for their unbiased HEX.

The CEE countries Czechia, Estonia, and Poland also did not follow the expected pattern in terms of educational differences in survey participation. Contrary to what is generally found in the literature, high-educated individuals were underrepresented in all three countries, most profoundly so in Czechia. One explanation for this curious finding is that in all three countries, high-educated individuals are much more likely to keep working at older ages, presumably due to low pension replacement rates. This pattern holds for both men and women. For the age group of 65–74, Estonian academics had the highest employment rate in the sample (26.9%), followed by the highly educated in Czechia (20.5%), Italy (19.7%), and Poland (18.6%) [82]. As a result, the highly educated might be less likely to participate in surveys due to time constraints: when an interviewer knocks on their doors, they might simply be at work. A second, somewhat speculative, explanation for the low participation of high-educated individuals in Czechia, Estonia, and Poland could be related to trust or the lack thereof. It is well established that post-communist societies in Europe have, on average, lower levels of trust in institutions [83] and lower levels of social trust [84]. If the highly educated were more distrustful than low-educated individuals, this could explain the participation pattern in the three countries. What contradicts this speculation is the fact that Slovenia is also a CEE country with a similar history. However, the Slovenian SHARE data follow the common pattern of too few low-educated respondents.

HEX is calculated by combining the prevalence of good and bad health from survey data with life tables. This study analysed how distortion in the education structure of surveys affects HEX via biases in prevalence rates. In addition, one could analyse whether educational differences in life expectancy also add to the bias. Due to data restrictions, it is commonly assumed that all educational groups share the same life expectancies when applying Sullivan’s method. However, Eurostat data for a small sample of European countries show that all countries but Italy have a clear education gradient in life expectancy. The educational differences are most pronounced in the CEE countries, save Slovenia, and are weakest in the Nordic countries [13]. If and how these differences bias HEX in the context of distorted surveys cannot be said a priori, as the bias depends on the interactions between the education distribution in the general population and the education-related response behaviour in the respective country. Thus, this study only focused on distortions due to prevalence rates to stay within scope. Furthermore, this study evaluated HEX in its most common form, which is without education-specific mortality. However, future studies should explore how educational differences in life expectancy affect the bias in HEX, especially in countries where the education gradient in mortality is strong.

The main limitation of this paper is data driven. For most countries, SHARE captures non-institutionalised persons only. Since the census does not differentiate between institutionalised and non-institutionalised persons, it was assumed that both groups are comparable. If this assumption is violated due to educational differences between the two groups, prevalence rates based on EW might deviate from the prevalence rates for the general population.

Overall, the findings of this study highlight the need to account for distortions in the education structure of survey data. First and foremost, this can be achieved by preventing the misrepresentation of certain educational groups in the first place, and if prevention does not lead to accurate representation, by adjusting for deviations with survey methods such as calibrated weights. Literature has shown that survey modes [23], recruitment methods [85], interviewer experience, and the number of attempted contacts [22] affect survey participation and consequently might be helpful for counteracting heterogeneities in survey representation. However, past evidence has also revealed that response rates have declined over time [22], and that the gap in response behaviour between high- and low-educated individuals has increased [6]. If this pattern continues, survey methods that adjust for misrepresentation will become even more important in the future. Although auxiliary information on the education structure in the general population is not available for each European country at any given year, censuses might still allow for the calibration of weights since the education structure at old age changes slowly [86], or as Schumacher [87] puts it: “education does not ‘jump’”.

## Conclusion

Survey participation differs substantially among educational groups, which leads to biased health expectancy (HEX) when the discrepancies are not accounted for. This study was the first to explore the magnitude and direction of the bias in HEX for 13 European countries based on the Survey of Health, Ageing and Retirement in Europe (SHARE) for 2011. To this end, calibrated weights were generated so that the education structure in SHARE would resemble that of the respective Population and Housing Census.

The analysis revealed that SHARE did not accurately resemble the education structure in the general population for 11 of the 13 countries investigated, which lead to substantial biases in HEX. In most of the datasets, high-educated individuals were overrepresented. Due to the positive correlation between educational attainment and good health, HEX was upward-biased for these countries by as much as 0.87 years. Remarkably, most CEE countries showed the opposite pattern that high-educated individuals were underrepresented. As a result, HEX was underestimated for these countries by up to 0.85 years.

Understanding the sensitivity of HEX measures is crucial because of their immense scientific and political influence. In the context of ever decreasing survey response rates, it is of utmost importance that the flawed education structure in survey data is prevented and adjusted for. Only then, it is possible to accurately assess policy targets based on HEX.

## Appendix

### 1.1 Proportions in SHARE versus those in the censuses

See Tables 1, 2, 3, 4, 5, 6, 7, 8, 9, 10, 11, 12, 13.

**Table 1:** Austria

Age	Education	Men				Women			
		SHARE		Census		SHARE		Census	
		*N*	%	*N*	%	*N*	%	*N*	%
50–59	Low	63	9.5	86,887	15.4	206	23.9	170,957	29.6
	Medium	405	61.3	367,802	65.0	394	45.8	326,967	56.6
	High	187	28.3	111,220	19.7	240	27.9	79,609	13.8
	Unknown	6	0.9	0	0.0	21	2.4	0	0.0
	Total	661	100.0	565,909	100.0	861	100.0	577,533	100.0
60–69	Low	98	13.0	79,259	18.8	255	25.4	176,335	38.1
	Medium	416	55.2	263,463	62.6	519	51.7	249,273	53.9
	High	230	30.5	78,097	18.6	218	21.7	37,067	8.0
	Unknown	10	1.3	0	0.0	11	1.1	0	0.0
	Total	754	100.0	420,819	100.0	1003	100.0	462,675	100.0
70–79	Low	92	16.5	86,735	29.0	316	43.3	215,302	57.6
	Medium	284	51.0	164,705	55.1	272	37.3	143,121	38.3
	High	176	31.6	47,386	15.9	132	18.1	15,268	4.1
	Unknown	5	0.9	0	0.0	10	1.4	0	0.0
	Total	557	100.0	298,826	100.0	730	100.0	373,691	100.0
80–89	Low	47	25.1	41,385	33.6	152	50.5	151,359	63.9
	Medium	81	43.3	64,003	51.9	103	34.2	77,106	32.6
	High	51	27.3	17,831	14.5	41	13.6	8221	3.5
	Unknown	8	4.3	0	0.0	5	1.7	0	0.0
	Total	187	100.0	123,219	100.0	301	100.0	236,686	100.0
90 +	Low	7	35.0	4742	36.4	20	58.8	29,223	66.7
	Medium	6	30.0	6016	46.2	11	32.4	12,972	29.6
	High	6	30.0	2262	17.4	2	5.9	1647	3.8
	Unknown	1	5.0	0	0.0	1	2.9	0	0.0
	Total	20	100.0	13,020	100.0	34	100.0	43,842	100.0

**Table 2:** Belgium

Age	Education	Men				Women			
		SHARE		Census		SHARE		Census	
		*N*	%	*N*	%	*N*	%	*N*	%
50–59	Low	298	35.7	295,514	39.9	329	31.1	296,759	40.0
	Medium	217	26.0	210,435	28.4	339	32.0	213,803	28.8
	High	297	35.6	180,721	24.4	364	34.4	183,135	24.7
	Unknown	23	2.8	54,628	7.4	26	2.5	48,576	6.5
	Total	835	100.0	741,298	100.0	1,058	100.0	742,273	100.0
60–69	Low	299	38.4	264,576	48.0	331	40.4	315,593	54.4
	Medium	203	26.1	122,045	22.2	240	29.3	117,672	20.3
	High	265	34.0	121,519	22.1	236	28.8	102,593	17.7
	Unknown	12	1.5	42,791	7.8	13	1.6	44,314	7.6
	Total	779	100.0	550,931	100.0	820	100.0	580,172	100.0
70–79	Low	213	46.1	223,675	59.3	294	53.0	312,619	66.1
	Medium	103	22.3	58,576	15.5	131	23.6	64,268	13.6
	High	142	30.7	56,867	15.1	122	22.0	44,972	9.5
	Unknown	4	0.9	37,802	10.0	8	1.4	51,189	10.8
	Total	462	100.0	376,920	100.0	555	100.0	473,048	100.0
80–89	Low	140	56.5	106,684	61.5	247	69.0	217,454	69.8
	Medium	50	20.2	25,946	14.9	59	16.5	34,466	11.1
	High	54	21.8	20,467	11.8	50	14.0	18,623	6.0
	Unknown	4	1.6	20,457	11.8	2	0.6	41,186	13.2
	Total	248	100.0	173,554	100.0	358	100.0	311,729	100.0
90 +	Low	16	64.0	9905	61.3	42	73.7	35,935	69.7
	Medium	6	24.0	2155	13.3	6	10.5	4791	9.3
	High	2	8.0	2004	12.4	8	14.0	3018	5.9
	Unknown	1	4.0	2087	12.9	1	1.8	7835	15.2
	Total	25	100.0	16,151	100.0	57	100.0	51,579	100.0

**Table 3:** Czechia

Age	Education	Men				Women			
		SHARE		Census		SHARE		Census	
		*N*	%	*N*	%	*N*	%	*N*	%
50–59	Low	284	45.4	60,953	8.8	373	42.4	143,319	20.0
	Medium	244	39.0	495,476	71.2	397	45.1	468,487	65.5
	High	93	14.9	108,342	15.6	98	11.1	82,322	11.5
	Unknown	5	0.8	31,312	4.5	12	1.4	20,992	2.9
	Total	626	100.0	696,083	100.0	880	100.0	715,120	100.0
60–69	Low	423	46.0	62,905	10.4	545	43.8	180,716	25.9
	Medium	360	39.1	443,380	73.0	558	44.8	441,352	63.3
	High	117	12.7	84,381	13.9	122	9.8	59,052	8.5
	Unknown	20	2.2	16,975	2.8	20	1.6	16,155	2.3
	Total	920	100.0	607,641	100.0	1,245	100.0	697,275	100.0
70–79	Low	219	41.5	47,015	16.4	372	53.6	173,996	42.4
	Medium	205	38.8	190,935	66.6	249	35.9	202,787	49.4
	High	94	17.8	41,874	14.6	62	8.9	22,715	5.5
	Unknown	10	1.9	6933	2.4	11	1.6	11,118	2.7
	Total	528	100.0	286,757	100.0	694	100.0	410,616	100.0
80–89	Low	76	39.4	23,055	20.0	181	63.7	120,760	50.6
	Medium	69	35.8	69,424	60.3	77	27.1	100,546	42.1
	High	44	22.8	19,280	16.7	19	6.7	8,445	3.5
	Unknown	4	2.1	3399	3.0	7	2.5	8,933	3.7
	Total	193	100.0	115,158	100.0	284	100.0	238,684	100.0
90 +	Low	4	33.3	1816	23.0	14	51.9	13,684	54.6
	Medium	3	25.0	4571	57.9	11	40.7	9,393	37.5
	High	4	33.3	1158	14.7	1	3.7	736	2.9
	Unknown	1	8.3	352	4.5	1	3.7	1,242	5.0
	Total	12	100.0	7897	100.0	27	100.0	25,055	100.0

**Table 4:** Denmark

Age	Education	Men				Women			
		SHARE		Census		SHARE		Census	
		*N*	%	*N*	%	*N*	%	*N*	%
50–59	Low	40	10.5	86,106	24.0	58	13.1	100,625	28.2
	Medium	177	46.3	172,014	47.9	126	28.4	131,424	36.8
	High	158	41.4	91,671	25.5	255	57.6	117,706	32.9
	Unknown	7	1.8	9572	2.7	4	0.9	7650	2.1
	Total	382	100.0	359,363	100.0	443	100.0	357,405	100.0
60–69	Low	33	9.6	92,455	27.4	54	14.7	124,807	36.1
	Medium	168	48.8	155,927	46.3	130	35.3	135,091	39.1
	High	136	39.5	82,314	24.4	179	48.6	80,054	23.1
	Unknown	7	2.0	6145	1.8	5	1.4	5932	1.7
	Total	344	100.0	336,841	100.0	368	100.0	345,884	100.0
70–79	Low	36	17.8	67,694	37.9	77	35.3	112,258	54.0
	Medium	101	50.0	72,763	40.8	77	35.3	60,975	29.3
	High	64	31.7	33,064	18.5	61	28.0	29,855	14.3
	Unknown	1	0.5	4901	2.7	3	1.4	4969	2.4
	Total	202	100.0	178,422	100.0	218	100.0	208,057	100.0
80–89	Low	16	16.8	35,204	48.7	74	50.0	78,481	66.6
	Medium	41	43.2	23,873	33.0	48	32.4	25,763	21.9
	High	33	34.7	11,782	16.3	25	16.9	11,554	9.8
	Unknown	5	5.3	1437	2.0	1	0.7	2045	1.7
	Total	95	100.0	72,296	100.0	148	100.0	117,843	100.0
90 +	Low	4	30.8	335	3.5	15	60.0	1263	4.4
	Medium	5	38.5	166	1.7	8	32.0	309	1.1
	High	3	23.1	278	2.9	1	4.0	190	0.7
	Unknown	1	7.7	8912	92.0	1	4.0	26,913	93.9
	Total	13	100.0	9691	100.0	25	100.0	28,675	100.0

**Table 5:** Estonia

Age	Education	Men				Women			
		SHARE		Census		SHARE		Census	
		*N*	%	*N*	%	*N*	%	*N*	%
50–59	Low	156	19.5	6936	8.5	137	12.6	5282	5.5
	Medium	481	60.1	47,118	57.8	628	57.9	46,585	48.3
	High	162	20.3	26,085	32.0	318	29.3	43,609	45.2
	Unknown	1	0.1	1425	1.7	1	0.1	921	1.0
	Total	800	100.0	81,564	100.0	1,084	100.0	96,397	100.0
60–69	Low	278	31.2	9704	17.0	232	19.8	11,609	14.4
	Medium	419	47.0	29,786	52.3	696	59.4	40,115	49.8
	High	193	21.7	16,698	29.3	242	20.7	28,206	35.0
	Unknown	1	0.1	779	1.4	1	0.1	688	0.9
	Total	891	100.0	56,967	100.0	1171	100.0	80,618	100.0
70–79	Low	318	41.6	11,188	28.9	476	39.6	24,889	33.4
	Medium	281	36.7	16,107	41.6	483	40.1	28,996	38.9
	High	165	21.6	10,877	28.1	243	20.2	19,706	26.5
	Unknown	1	0.1	509	1.3	1	0.1	882	1.2
	Total	765	100.0	38,681	100.0	1203	100.0	74,473	100.0
80–89	Low	147	52.9	5698	42.8	295	57.4	20,559	51.9
	Medium	75	27.0	4154	31.2	157	30.5	11,561	29.2
	High	55	19.8	3230	24.3	61	11.9	6599	16.6
	Unknown	1	0.4	220	1.7	1	0.2	916	2.3
	Total	278	100.0	13,302	100.0	514	100.0	39,635	100.0
90 +	Low	7	53.8	441	48.3	31	67.4	2893	62.3
	Medium	3	23.1	277	30.3	11	23.9	1114	24.0
	High	2	15.4	163	17.9	3	6.5	411	8.9
	Unknown	1	7.7	32	3.5	1	2.2	222	4.8
	Total	13	100.0	913	100.0	46	100.0	4640	100.0

**Table 6:** France

Age	Education	Men				Women			
		SHARE		Census		SHARE		Census	
		*N*	%	*N*	%	*N*	%	*N*	%
50–59	Low	181	22.5	1,303,815	31.3	304	30.2	1,703,720	38.8
	Medium	402	49.9	1,959,813	47.1	414	41.1	1,716,270	39.1
	High	203	25.2	895,551	21.5	262	26.0	969,392	22.1
	Unknown	20	2.5	144	0.0	28	2.8	113	0.0
	Total	806	100.0	4,159,323	100.0	1008	100.0	4,389,495	100.0
60–69	Low	284	34.4	1,264,695	40.0	406	41.8	1,748,789	51.3
	Medium	315	38.2	1,277,057	40.4	320	32.9	1,106,511	32.5
	High	201	24.4	617,162	19.5	220	22.6	552,731	16.2
	Unknown	25	3.0	51	0.0	26	2.7	29	0.0
	Total	825	100.0	3,158,965	100.0	972	100.0	3,408,060	100.0
70–79	Low	271	50.6	1,182,924	57.0	461	67.7	1,910,878	70.9
	Medium	166	31.0	645,923	31.1	130	19.1	576,136	21.4
	High	90	16.8	247,312	11.9	70	10.3	207,284	7.7
	Unknown	9	1.7	0	0.0	20	2.9	0	0.0
	Total	536	100.0	2,076,159	100.0	681	100.0	2,694,298	100.0
80–89	Low	194	69.5	712,663	68.2	368	79.7	1,476,693	78.0
	Medium	52	18.6	220,702	21.1	52	11.3	291,174	15.4
	High	27	9.7	111,301	10.7	30	6.5	125,780	6.6
	Unknown	6	2.2	0	0.0	12	2.6	0	0.0
	Total	279	100.0	1,044,666	100.0	462	100.0	1,893,647	100.0
90 +	Low	15	53.6	80,282	67.6	60	85.7	277,819	74.4
	Medium	7	25.0	23,167	19.5	4	5.7	59,599	16.0
	High	5	17.9	15,255	12.9	5	7.1	35,760	9.6
	Unknown	1	3.6	0	0.0	1	1.4	0	0.0
	Total	28	100.0	118,704	100.0	70	100.0	373,178	100.0

**Table 7:** Germany

Age	Education	Men				Women			
		SHARE		Census		SHARE		Census	
		*N*	%	*N*	%	*N*	%	*N*	%
50–59	Low	5	4.8	662,600	11.6	22	11.6	1,061,130	18.2
	Medium	53	51.0	3,137,380	54.7	103	54.5	3,164,500	54.4
	High	41	39.4	1,936,590	33.8	54	28.6	1,590,890	27.4
	Unknown	5	4.8	0	0.0	10	5.3	0	0.0
	Total	104	100.0	5,736,570	100.0	189	100.0	5,816,520	100.0
60–69	Low	13	4.4	531,050	12.4	41	12.7	1,184,640	26.0
	Medium	160	54.2	2,256,210	52.8	176	54.5	2,468,540	54.1
	High	106	35.9	1,486,110	34.8	98	30.3	907,790	19.9
	Unknown	16	5.4	0	0.0	8	2.5	0	0.0
	Total	295	100.0	4,273,370	100.0	323	100.0	4,560,970	100.0
70–79	Low	10	3.7	609,250	16.7	56	23.4	1,936,480	43.3
	Medium	152	55.9	1,983,600	54.2	141	59.0	2,023,110	45.2
	High	100	36.8	1,064,890	29.1	38	15.9	513,770	11.5
	Unknown	10	3.7	0	0.0	4	1.7	0	0.0
	Total	272	100.0	3,657,740	100.0	239	100.0	4,473,360	100.0
80–89	Low	5	6.0	246,230	20.1	39	41.9	1,278,640	54.4
	Medium	47	56.6	656,190	53.5	36	38.7	884,140	37.6
	High	29	34.9	325,090	26.5	15	16.1	189,760	8.1
	Unknown	2	2.4	0	0.0	3	3.2	0	0.0
	Total	83	100.0	1,227,510	100.0	93	100.0	2,352,540	100.0
90 +	Low	1	20.0	21,300	19.7	3	25.0	225,740	55.8
	Medium	2	40.0	56,130	52.0	6	50.0	149,430	37.0
	High	1	20.0	30,450	28.2	2	16.7	29,180	7.2
	Unknown	1	20.0	0	0.0	1	8.3	0	0.0
	Total	5	100.0	107,880	100.0	12	100.0	404,350	100.0

**Table 8:** Hungary

Age	Education	Men				Women			
		SHARE		Census		SHARE		Census	
		*N*	%	*N*	%	*N*	%	*N*	%
50–59	Low	52	12.3	120,662	17.8	152	27.3	217,215	28.6
	Medium	309	72.9	453,647	66.8	323	58.1	406,335	53.5
	High	62	14.6	104,882	15.4	80	14.4	135,941	17.9
	Unknown	1	0.2	0	0.0	1	0.2	0	0.0
	Total	424	100.0	679,191	100.0	556	100.0	759,491	100.0
60–69	Low	93	17.9	125,036	24.3	200	33.3	271,885	41.1
	Medium	318	61.3	293,669	57.0	296	49.3	297,272	44.9
	High	107	20.6	96,653	18.8	104	17.3	92,447	14.0
	Unknown	1	0.2	0	0.0	1	0.2	0	0.0
	Total	519	100.0	515,358	100.0	601	100.0	661,604	100.0
70–79	Low	79	29.5	177,620	63.8	203	55.9	352,237	73.9
	Medium	133	49.6	52,768	18.9	117	32.2	88,451	18.6
	High	55	20.5	48,165	17.3	42	11.6	35,676	7.5
	Unknown	1	0.4	0	0.0	1	0.3	0	0.0
	Total	268	100.0	278,553	100.0	363	100.0	476,364	100.0
80–89	Low	39	41.1	68,943	64.7	118	77.1	212,204	84.8
	Medium	37	38.9	17,325	16.3	25	16.3	25,654	10.3
	High	18	18.9	20,313	19.1	9	5.9	12,365	4.9
	Unknown	1	1.1	0	0.0	1	0.7	0	0.0
	Total	95	100.0	106,581	100.0	153	100.0	250,223	100.0
90 +	Low	4	44.4	7092	67.5	12	60.0	27,893	87.4
	Medium	2	22.2	1606	15.3	6	30.0	2657	8.3
	High	2	22.2	1806	17.2	1	5.0	1374	4.3
	Unknown	1	11.1	0	0.0	1	5.0	0	0.0
	Total	9	100.0	10,504	100.0	20	100.0	31,924	100.0

**Table 9:** Italy

Age	Education	Men				Women			
		SHARE		Census		SHARE		Census	
		*N*	%	*N*	%	*N*	%	*N*	%
50–59	Low	169	46.8	1,896,312	49.5	280	55.2	2,072,038	51.3
	Medium	156	43.2	1,453,862	37.9	167	32.9	1,462,737	36.2
	High	32	8.9	484,544	12.6	51	10.1	502,340	12.4
	Unknown	4	1.1	0	0.0	9	1.8	0	0.0
	Total	361	100.0	3,834,718	100.0	507	100.0	4,037,115	100.0
60–69	Low	346	60.6	2,079,003	63.3	516	73.6	2,586,617	72.4
	Medium	171	29.9	874,563	26.6	135	19.3	711,707	19.9
	High	40	7.0	333,239	10.1	41	5.8	275,036	7.7
	Unknown	14	2.5	0	0.0	9	1.3	0	0.0
	Total	571	100.0	3,286,805	100.0	701	100.0	3,573,360	100.0
70–79	Low	384	78.9	1,972,475	78.6	413	81.1	2,684,196	86.1
	Medium	69	14.2	374,245	14.9	68	13.4	336,083	10.8
	High	30	6.2	161,577	6.4	19	3.7	95,823	3.1
	Unknown	4	0.8	0	0.0	9	1.8	0	0.0
	Total	487	100.0	2,508,297	100.0	509	100.0	3,116,102	100.0
80–89	Low	144	83.7	936,638	82.8	165	93.2	1,778,669	89.4
	Medium	14	8.1	125,891	11.1	9	5.1	161,484	8.1
	High	11	6.4	68,965	6.1	2	1.1	48,485	2.4
	Unknown	3	1.7	0	0.0	1	0.6	0	0.0
	Total	172	100.0	1,131,494	100.0	177	100.0	1,988,638	100.0
90 +	Low	18	85.7	110,847	83.4	27	87.1	354,613	91.5
	Medium	1	4.8	12,692	9.5	2	6.5	24,650	6.4
	High	1	4.8	9432	7.1	1	3.2	8174	2.1
	Unknown	1	4.8	0	0.0	1	3.2	0	0.0
	Total	21	100.0	132,971	100.0	31	100.0	387,437	100.0

**Table 10:** Poland

Age	Education	Men				Women			
		SHARE		Census		SHARE		Census	
		*N*	%	*N*	%	*N*	%	*N*	%
50–59	Low	29	16.7	421,166	15.0	57	20.9	478,116	16.1
	Medium	115	66.1	1,981,997	70.8	156	57.1	2,018,930	67.8
	High	11	6.3	330,327	11.8	21	7.7	423,912	14.2
	Unknown	19	10.9	67,063	2.4	39	14.3	57,925	1.9
	Total	174	100.0	2,800,553	100.0	273	100.0	2,978,883	100.0
60–69	Low	73	22.6	420,733	24.7	144	38.9	672,145	32.7
	Medium	161	49.8	1,019,057	59.9	190	51.4	1,116,799	54.3
	High	39	12.1	230,425	13.5	15	4.1	238,273	11.6
	Unknown	50	15.5	31,166	1.8	21	5.7	29,409	1.4
	Total	323	100.0	1,701,381	100.0	370	100.0	2,056,626	100.0
70–79	Low	80	46.0	395,289	40.8	136	66.0	843,444	55.3
	Medium	57	32.8	432,775	44.7	51	24.8	543,307	35.6
	High	19	10.9	125,120	12.9	7	3.4	113,995	7.5
	Unknown	18	10.3	14,640	1.5	12	5.8	23,721	1.6
	Total	174	100.0	967,824	100.0	206	100.0	1,524,467	100.0
80–89	Low	47	60.3	199,977	53.3	79	75.2	619,859	73.2
	Medium	21	26.9	120,999	32.3	10	9.5	170,244	20.1
	High	5	6.4	47,888	12.8	2	1.9	32,531	3.8
	Unknown	5	6.4	6312	1.7	14	13.3	24,220	2.9
	Total	78	100.0	375,176	100.0	105	100.0	846,854	100.0
90 +	Low	3	50.0	17,756	62.4	13	81.3	73,860	77.2
	Medium	1	16.7	7120	25.0	1	6.3	14,091	14.7
	High	1	16.7	2691	9.5	1	6.3	2219	2.3
	Unknown	1	16.7	891	3.1	1	6.3	5478	5.7
	Total	6	100.0	28,458	100.0	16	100.0	95,648	100.0

**Table 11:** Portugal

Age	Education	Men				Women			
		SHARE		Census		SHARE		Census	
		*N*	%	*N*	%	*N*	%	*N*	%
50–59	Low	184	69.7	517,091	77.4	285	74.4	558,254	76.3
	Medium	35	13.3	79,694	11.9	46	12.0	79,177	10.8
	High	40	15.2	71,558	10.7	48	12.5	94,237	12.9
	Unknown	5	1.9	0	0.0	4	1.0	0	0.0
	Total	264	100.0	668,343	100.0	383	100.0	731,668	100.0
60–69	Low	258	78.2	469,350	85.1	299	77.5	556,689	87.7
	Medium	40	12.1	38,466	7.0	33	8.5	29,058	4.6
	High	30	9.1	43,734	7.9	37	9.6	49,145	7.7
	Unknown	2	0.6	0	0.0	17	4.4	0	0.0
	Total	330	100.0	551,550	100.0	386	100.0	634,892	100.0
70–79	Low	158	78.6	364,241	90.9	181	86.2	493,050	93.8
	Medium	16	8.0	16,569	4.1	6	2.9	12,310	2.3
	High	23	11.4	19,782	4.9	15	7.1	20,192	3.8
	Unknown	4	2.0	0	0.0	8	3.8	0	0.0
	Total	201	100.0	400,592	100.0	210	100.0	525,552	100.0
80–89	Low	49	77.8	155,428	92.0	92	82.9	279,326	95.2
	Medium	5	7.9	6162	3.6	8	7.2	6897	2.4
	High	4	6.3	7370	4.4	7	6.3	7061	2.4
	Unknown	5	7.9	0	0.0	4	3.6	0	0.0
	Total	63	100.0	168,960	100.0	111	100.0	293,284	100.0
90 +	Low	4	57.1	18,068	91.4	6	60.0	48,108	95.8
	Medium	1	14.3	748	3.8	1	10.0	1109	2.2
	High	1	14.3	952	4.8	1	10.0	990	2.0
	Unknown	1	14.3	0	0.0	2	20.0	0	0.0
	Total	7	100.0	19,768	100.0	10	100.0	50,207	100.0

**Table 12:** Slovenia

Age	Education	Men				Women			
		SHARE		Census		SHARE		Census	
		*N*	%	*N*	%	*N*	%	*N*	%
50–59	Low	87	20.8	39,279	25.3	152	29.1	51,986	34.8
	Medium	270	64.4	92,682	59.7	263	50.4	71,200	47.6
	High	61	14.6	23,315	15.0	106	20.3	26,313	17.6
	Unknown	1	0.2	0	0.0	1	0.2	0	0.0
	Total	419	100.0	155,276	100.0	522	100.0	149,499	100.0
60–69	Low	61	16.0	26,630	25.5	167	36.9	51,794	45.9
	Medium	239	62.7	60,974	58.3	204	45.0	46,809	41.5
	High	79	20.7	17,011	16.3	81	17.9	14,298	12.7
	Unknown	2	0.5	0	0.0	1	0.2	0	0.0
	Total	381	100.0	104,615	100.0	453	100.0	112,901	100.0
70–79	Low	91	32.5	20,867	31.6	206	59.2	59,259	63.2
	Medium	134	47.9	35,849	54.3	108	31.0	28,520	30.4
	High	52	18.6	9365	14.2	33	9.5	6036	6.4
	Unknown	3	1.1	0	0.0	1	0.3	0	0.0
	Total	280	100.0	66,081	100.0	348	100.0	93,815	100.0
80–89	Low	42	38.2	8192	36.2	114	63.7	36,409	67.1
	Medium	45	40.9	10,734	47.4	55	30.7	15,386	28.4
	High	22	20.0	3729	16.5	9	5.0	2434	4.5
	Unknown	1	0.9	0	0.0	1	0.6	0	0.0
	Total	110	100.0	22,655	100.0	179	100.0	54,229	100.0
90 +	Low	1	25.0	608	36.4	17	85.0	4361	67.1
	Medium	1	25.0	751	45.0	1	5.0	1877	28.9
	High	1	25.0	310	18.6	1	5.0	266	4.1
	Unknown	1	25.0	0	0.0	1	5.0	0	0.0
	Total	4	100.0	1669	100.0	20	100.0	6504	100.0

**Table 13:** Spain

Age	Education	Men				Women			
		SHARE		Census		SHARE		Census	
		*N*	%	*N*	%	*N*	%	*N*	%
50–59	Low	252	62.1	1,644,040	55.9	347	63.2	1,807,090	60.4
	Medium	77	19.0	585,055	19.9	105	19.1	555,465	18.6
	High	63	15.5	711,115	24.2	71	12.9	627,870	21.0
	Unknown	14	3.4	0	0.0	26	4.7	0	0.0
	Total	406	100.0	2,940,210	100.0	549	100.0	2,990,425	100.0
60–69	Low	370	72.5	1,522,130	68.2	467	82.8	1,900,160	78.8
	Medium	52	10.2	279,630	12.5	32	5.7	241,585	10.0
	High	53	10.4	428,610	19.2	38	6.7	268,510	11.1
	Unknown	35	6.9	0	0.0	27	4.8	0	0.0
	Total	510	100.0	2,230,370	100.0	564	100.0	2,410,255	100.0
70–79	Low	401	84.6	1,253,700	80.2	458	88.8	1,763,050	89.3
	Medium	26	5.5	115,365	7.4	19	3.7	105,125	5.3
	High	28	5.9	193,660	12.4	17	3.3	106,470	5.4
	Unknown	19	4.0	0	0.0	22	4.3	0	0.0
	Total	474	100.0	1,562,725	100.0	516	100.0	1,974,645	100.0
80–89	Low	209	87.1	663,570	85.5	292	91.0	1,185,560	92.4
	Medium	5	2.1	41,485	5.3	3	0.9	49,605	3.9
	High	15	6.3	70,815	9.1	11	3.4	48,465	3.8
	Unknown	11	4.6	0	0.0	15	4.7	0	0.0
	Total	240	100.0	775,870	100.0	321	100.0	1,283,630	100.0
90 +	Low	25	83.3	80,655	84.0	54	94.7	226,135	91.9
	Medium	2	6.7	6185	6.4	1	1.8	9610	3.9
	High	1	3.3	9170	9.6	1	1.8	10,450	4.2
	Unknown	2	6.7	0	0.0	1	1.8	0	0.0
	Total	30	100.0	96,010	100.0	57	100.0	246,195	100.0

### 1.2 Prevalence rates and HEX based on GALI by weighting strategy

See Tables 14, 15, 16, 17, 18, 19, 20, 21, 22, 23, 24, 25, 26.

**Table 14:** Austria

Gender	Age	Replicated weights						Education-adjusted weights						Bias			
		$\pi$	95% CI		HEX	95% CI		$\pi$	95% CI		HEX	95% CI		∆HEX	95% CI		∆%
Men	50–54	0.11	0.07	0.15	25.88	25.15	26.60	0.13	0.08	0.17	25.54	24.80	26.29	0.33	0.27	0.39	1.30
	55–59	0.14	0.10	0.19	21.99	21.27	22.71	0.17	0.11	0.22	21.73	20.99	22.46	0.26	0.20	0.32	1.20
	60–64	0.12	0.08	0.15	18.45	17.73	19.18	0.13	0.09	0.17	18.29	17.56	19.03	0.16	0.11	0.21	0.86
	65–69	0.10	0.07	0.13	15.07	14.31	15.82	0.10	0.07	0.13	14.97	14.20	15.73	0.10	0.04	0.16	0.66
	70–74	0.17	0.13	0.21	11.75	10.95	12.56	0.18	0.13	0.22	11.64	10.82	12.47	0.11	0.05	0.17	0.94
	75–79	0.14	0.09	0.19	8.87	7.98	9.76	0.16	0.10	0.22	8.79	7.88	9.70	0.08	− 0.02	0.17	0.86
	80–84	0.23	0.15	0.30	6.20	5.12	7.27	0.23	0.15	0.31	6.23	5.14	7.33	− 0.04	− 0.17	0.10	− 0.61
	85 +	0.28	0.18	0.39	4.39	2.85	5.94	0.27	0.16	0.37	4.48	2.92	6.05	− 0.09	− 0.34	0.16	− 2.02
Women	50–54	0.06	0.03	0.08	29.34	28.81	29.87	0.06	0.03	0.09	28.94	28.40	29.48	0.40	0.36	0.43	1.37
	55–59	0.11	0.07	0.14	24.95	24.42	25.47	0.12	0.08	0.16	24.58	24.04	25.11	0.37	0.33	0.40	1.50
	60–64	0.09	0.06	0.11	20.91	20.40	21.43	0.09	0.07	0.12	20.60	20.07	21.12	0.32	0.28	0.35	1.53
	65–69	0.09	0.06	0.12	16.90	16.38	17.42	0.10	0.07	0.13	16.61	16.08	17.14	0.29	0.26	0.33	1.76
	70–74	0.17	0.13	0.20	13.03	12.51	13.56	0.18	0.14	0.21	12.76	12.22	13.29	0.28	0.24	0.31	2.17
	75–79	0.24	0.19	0.30	9.63	9.09	10.16	0.25	0.19	0.31	9.37	8.82	9.91	0.26	0.21	0.31	2.77
	80–84	0.26	0.20	0.33	6.84	6.30	7.37	0.29	0.22	0.36	6.61	6.07	7.15	0.23	0.17	0.28	3.42
	85 +	0.37	0.28	0.45	4.60	4.00	5.20	0.39	0.30	0.48	4.45	3.84	5.05	0.15	0.08	0.23	3.41

**Table 15:** Belgium

Gender	Age	Replicated weights						Education-adjusted weights						Bias			
		$\pi$	95% CI		HEX	95% CI		$\pi$	95% CI		HEX	95% CI		∆HEX	95% CI		∆%
Men	50–54	0.08	0.04	0.13	24.82	24.05	25.58	0.15	0.04	0.25	24.43	23.64	25.21	0.39	0.33	0.45	1.59
	55–59	0.16	0.12	0.19	20.77	20.00	21.53	0.17	0.13	0.21	20.68	19.90	21.46	0.08	0.03	0.13	0.41
	60–64	0.16	0.12	0.19	17.30	16.52	18.08	0.17	0.12	0.21	17.27	16.48	18.06	0.03	− 0.02	0.08	0.16
	65–69	0.14	0.10	0.18	14.01	13.20	14.82	0.14	0.10	0.18	14.05	13.23	14.88	− 0.04	− 0.11	0.02	− 0.29
	70–74	0.15	0.10	0.19	10.78	9.92	11.65	0.14	0.09	0.18	10.85	9.98	11.73	− 0.07	− 0.15	0.01	− 0.65
	75–79	0.25	0.19	0.30	7.76	6.80	8.72	0.24	0.18	0.30	7.78	6.81	8.76	− 0.03	− 0.12	0.06	− 0.37
	80–84	0.28	0.21	0.36	5.46	4.30	6.62	0.28	0.21	0.36	5.45	4.27	6.64	0.01	− 0.13	0.14	0.10
	85 +	0.39	0.30	0.48	3.71	2.02	5.40	0.39	0.29	0.50	3.70	1.97	5.43	0.01	− 0.21	0.24	0.32
Women	50–54	0.19	0.13	0.25	25.56	24.97	26.14	0.26	0.16	0.36	24.69	24.09	25.29	0.87	0.83	0.90	3.51
	55–59	0.20	0.17	0.24	21.84	21.26	22.41	0.22	0.17	0.27	21.33	20.75	21.91	0.51	0.47	0.54	2.38
	60–64	0.19	0.16	0.23	18.32	17.76	18.88	0.22	0.17	0.27	17.87	17.31	18.44	0.45	0.41	0.48	2.50
	65–69	0.23	0.18	0.27	14.83	14.28	15.37	0.25	0.19	0.30	14.50	13.95	15.05	0.32	0.28	0.36	2.23
	70–74	0.26	0.21	0.31	11.57	11.04	12.10	0.28	0.22	0.34	11.34	10.81	11.88	0.23	0.18	0.27	2.00
	75–79	0.32	0.26	0.38	8.62	8.11	9.13	0.34	0.27	0.41	8.45	7.94	8.96	0.17	0.12	0.21	1.97
	80–84	0.35	0.29	0.41	6.16	5.67	6.64	0.36	0.29	0.42	6.10	5.61	6.58	0.06	0.02	0.11	1.00
	85 +	0.43	0.36	0.50	4.23	3.71	4.74	0.43	0.36	0.51	4.19	3.67	4.71	0.03	− 0.02	0.09	0.82

**Table 16:** Czechia

Gender	Age	Replicated weights						Education-adjusted weights						Bias			
		$\pi$	95% CI		HEX	95% CI		$\pi$	95% CI		HEX	95% CI		∆HEX	95% CI		∆%
Men	50–54	0.13	0.06	0.19	22.03	21.37	22.70	0.10	0.04	0.16	22.26	21.62	22.90	− 0.22	− 0.28	− 0.17	− 1.01
	55–59	0.19	0.15	0.24	18.34	17.68	18.99	0.20	0.14	0.25	18.44	17.81	19.08	− 0.11	− 0.15	− 0.06	− 0.57
	60–64	0.15	0.11	0.19	15.25	14.58	15.91	0.15	0.11	0.20	15.37	14.74	16.01	− 0.13	− 0.17	− 0.09	− 0.84
	65–69	0.15	0.11	0.19	12.21	11.51	12.91	0.13	0.09	0.17	12.36	11.69	13.04	− 0.15	− 0.20	− 0.10	− 1.21
	70–74	0.15	0.11	0.20	9.39	8.60	10.17	0.15	0.10	0.20	9.47	8.72	10.23	− 0.09	− 0.15	− 0.02	− 0.90
	75–79	0.24	0.18	0.31	6.67	5.75	7.59	0.24	0.17	0.31	6.76	5.87	7.65	− 0.09	− 0.17	0.00	− 1.32
	80–84	0.34	0.24	0.45	4.59	3.38	5.80	0.32	0.20	0.44	4.68	3.52	5.85	− 0.09	− 0.24	0.06	− 2.01
	85 +	0.37	0.22	0.51	3.30	1.34	5.25	0.37	0.19	0.54	3.30	1.43	5.18	− 0.01	− 0.31	0.30	− 0.16
Women	50–54	0.12	0.07	0.17	25.90	25.42	26.38	0.10	0.05	0.15	26.75	26.29	27.21	− 0.85	− 0.88	− 0.81	− 3.17
	55–59	0.15	0.11	0.19	21.84	21.39	22.30	0.14	0.09	0.18	22.58	22.14	23.02	− 0.74	− 0.77	− 0.71	− 3.27
	60–64	0.11	0.08	0.14	18.08	17.64	18.52	0.09	0.06	0.11	18.75	18.33	19.18	− 0.67	− 0.70	− 0.65	− 3.60
	65–69	0.15	0.11	0.18	14.24	13.80	14.68	0.13	0.10	0.17	14.83	14.40	15.25	− 0.59	− 0.61	− 0.56	− 3.95
	70–74	0.20	0.15	0.24	10.75	10.31	11.20	0.19	0.14	0.24	11.31	10.87	11.74	− 0.55	− 0.58	− 0.52	− 4.87
	75–79	0.28	0.22	0.34	7.67	7.22	8.12	0.24	0.18	0.30	8.24	7.81	8.67	− 0.57	− 0.61	− 0.54	− 6.95
	80–84	0.32	0.23	0.41	5.25	4.78	5.71	0.28	0.18	0.37	5.71	5.26	6.16	− 0.46	− 0.51	− 0.41	− 8.07
	85 +	0.44	0.34	0.55	3.44	2.91	3.97	0.38	0.27	0.49	3.83	3.32	4.35	− 0.39	− 0.46	− 0.33	− 10.25

**Table 17:** Denmark

Gender	Age	Replicated weights						Education-adjusted weights						Bias			
		$\pi$	95% CI		HEX	95% CI		$\pi$	95% CI		HEX	95% CI		∆HEX	95% CI		∆%
Men	50–54	0.07	0.03	0.11	25.93	25.04	26.83	0.11	0.05	0.17	25.41	24.48	26.33	0.53	0.43	0.62	2.08
	55–59	0.09	0.05	0.14	21.88	20.99	22.77	0.13	0.06	0.20	21.50	20.59	22.42	0.37	0.28	0.47	1.74
	60–64	0.06	0.02	0.10	18.12	17.22	19.03	0.08	0.02	0.13	17.95	17.02	18.87	0.18	0.07	0.28	0.98
	65–69	0.07	0.04	0.11	14.34	13.40	15.28	0.11	0.05	0.17	14.25	13.30	15.21	0.09	− 0.01	0.19	0.64
	70–74	0.12	0.06	0.19	10.85	9.84	11.86	0.12	0.06	0.19	10.92	9.90	11.94	− 0.07	− 0.20	0.07	− 0.60
	75–79	0.22	0.13	0.30	7.81	6.69	8.94	0.21	0.12	0.31	7.88	6.74	9.01	− 0.06	− 0.24	0.11	− 0.81
	80–84	0.21	0.10	0.31	5.59	4.22	6.96	0.16	0.07	0.26	5.66	4.28	7.04	− 0.07	− 0.33	0.19	− 1.23
	85 +	0.38	0.24	0.51	3.50	1.52	5.48	0.40	0.25	0.56	3.34	1.32	5.37	0.16	− 0.24	0.56	4.79
Women	50–54	0.08	0.04	0.11	29.18	28.54	29.81	0.11	0.05	0.17	28.87	28.22	29.52	0.31	0.25	0.37	1.07
	55–59	0.08	0.04	0.12	24.97	24.35	25.59	0.10	0.05	0.14	24.83	24.20	25.46	0.14	0.08	0.20	0.58
	60–64	0.09	0.05	0.13	20.95	20.34	21.55	0.11	0.05	0.17	20.87	20.25	21.48	0.08	0.02	0.14	0.38
	65–69	0.06	0.03	0.10	17.10	16.50	17.70	0.07	0.02	0.11	17.11	16.51	17.70	0.00	− 0.07	0.06	− 0.02
	70–74	0.12	0.06	0.17	13.26	12.66	13.87	0.10	0.05	0.16	13.30	12.70	13.90	− 0.03	− 0.11	0.04	− 0.26
	75–79	0.10	0.04	0.16	9.95	9.36	10.54	0.10	0.03	0.16	9.92	9.32	10.51	0.03	− 0.05	0.12	0.32
	80–84	0.20	0.12	0.28	6.90	6.27	7.53	0.21	0.12	0.31	6.86	6.23	7.49	0.04	− 0.05	0.14	0.64
	85 +	0.32	0.22	0.43	4.61	3.91	5.32	0.32	0.21	0.43	4.65	3.94	5.35	− 0.03	− 0.15	0.08	− 0.75

**Table 18:** Estonia

		Replicated weights						Education-adjusted weights						Bias			
Gender	Age	$\pi$	95% CI		HEX	95% CI		$\pi$	95% CI		HEX	95% CI		∆HEX	95% CI		∆%
Men	50–54	0.14	0.10	0.18	19.55	18.93	20.17	0.13	0.09	0.17	19.92	19.31	20.52	− 0.37	− 0.41	− 0.32	− 1.84
	55–59	0.18	0.14	0.21	16.12	15.50	16.74	0.16	0.12	0.20	16.46	15.85	17.07	− 0.34	− 0.38	− 0.30	− 2.09
	60–64	0.19	0.16	0.23	13.13	12.49	13.78	0.18	0.14	0.21	13.41	12.78	14.05	− 0.28	− 0.32	− 0.24	− 2.10
	65–69	0.22	0.17	0.26	10.42	9.72	11.12	0.20	0.16	0.24	10.64	9.96	11.33	− 0.22	− 0.27	− 0.18	− 2.11
	70–74	0.27	0.22	0.31	8.04	7.25	8.84	0.25	0.21	0.30	8.22	7.43	9.00	− 0.18	− 0.23	− 0.12	− 2.13
	75–79	0.31	0.26	0.37	5.92	4.95	6.88	0.30	0.25	0.36	6.05	5.10	7.00	− 0.13	− 0.21	− 0.06	− 2.19
	80–84	0.45	0.38	0.52	4.11	2.81	5.42	0.43	0.36	0.50	4.24	2.95	5.53	− 0.13	− 0.25	0.00	− 2.97
	85 +	0.40	0.28	0.51	3.33	1.13	5.53	0.38	0.26	0.50	3.43	1.27	5.60	− 0.11	− 0.47	0.26	− 3.13
Women	50–54	0.10	0.08	0.13	24.91	24.47	25.34	0.10	0.07	0.13	25.35	24.92	25.78	− 0.45	− 0.47	− 0.42	− 1.76
	55–59	0.15	0.12	0.18	20.80	20.38	21.22	0.13	0.10	0.16	21.23	20.81	21.64	− 0.43	− 0.45	− 0.40	− 2.01
	60–64	0.18	0.15	0.21	16.97	16.56	17.38	0.17	0.14	0.20	17.34	16.93	17.74	− 0.36	− 0.39	− 0.34	− 2.10
	65–69	0.17	0.14	0.21	13.41	13.02	13.81	0.16	0.13	0.20	13.74	13.34	14.13	− 0.32	− 0.35	− 0.30	− 2.35
	70–74	0.24	0.20	0.27	9.95	9.56	10.34	0.22	0.18	0.25	10.24	9.85	10.62	− 0.29	− 0.31	− 0.27	− 2.82
	75–79	0.37	0.33	0.42	6.92	6.53	7.31	0.36	0.32	0.40	7.14	6.75	7.53	− 0.22	− 0.24	− 0.19	− 3.05
	80–84	0.43	0.38	0.49	4.77	4.37	5.18	0.41	0.36	0.47	4.96	4.55	5.36	− 0.18	− 0.21	− 0.15	− 3.70
	85 +	0.53	0.46	0.60	3.18	2.71	3.64	0.51	0.44	0.59	3.29	2.83	3.76	− 0.12	− 0.16	− 0.07	− 3.52

**Table 19:** France

Gender	Age	Replicated weights						Education-adjusted weights						Bias			
		$\pi$	95% CI		HEX	95% CI		$\pi$	95% CI		HEX	95% CI		∆HEX	95% CI		∆%
Men	50–54	0.10	0.06	0.13	25.71	24.98	26.43	0.10	0.07	0.14	25.54	24.80	26.27	0.17	0.11	0.23	0.66
	55–59	0.10	0.07	0.13	21.85	21.12	22.58	0.11	0.08	0.14	21.73	20.99	22.47	0.13	0.08	0.17	0.58
	60–64	0.11	0.08	0.14	18.21	17.46	18.96	0.12	0.09	0.15	18.11	17.35	18.87	0.10	0.05	0.15	0.55
	65–69	0.12	0.08	0.15	14.70	13.92	15.48	0.12	0.08	0.16	14.63	13.84	15.41	0.07	0.02	0.13	0.51
	70–74	0.18	0.13	0.23	11.29	10.47	12.12	0.19	0.14	0.23	11.25	10.41	12.08	0.05	− 0.02	0.12	0.42
	75–79	0.19	0.14	0.24	8.31	7.41	9.21	0.20	0.15	0.25	8.29	7.39	9.20	0.02	− 0.06	0.10	0.24
	80–84	0.36	0.29	0.43	5.58	4.53	6.63	0.36	0.29	0.43	5.57	4.51	6.63	0.00	− 0.10	0.11	0.07
	85 +	0.43	0.33	0.53	3.92	2.51	5.33	0.43	0.33	0.53	3.94	2.51	5.36	− 0.02	− 0.20	0.17	− 0.40
Women	50–54	0.10	0.06	0.13	30.20	29.67	30.73	0.11	0.07	0.14	29.99	29.46	30.53	0.20	0.17	0.24	0.68
	55–59	0.10	0.08	0.13	26.05	25.54	26.57	0.11	0.08	0.14	25.90	25.38	26.42	0.15	0.12	0.19	0.60
	60–64	0.08	0.06	0.10	22.01	21.50	22.53	0.08	0.06	0.11	21.89	21.37	22.40	0.13	0.10	0.16	0.59
	65–69	0.11	0.08	0.14	17.91	17.40	18.42	0.12	0.09	0.16	17.79	17.27	18.30	0.12	0.09	0.16	0.68
	70–74	0.15	0.11	0.19	14.02	13.51	14.52	0.16	0.12	0.20	13.96	13.45	14.47	0.06	0.02	0.10	0.42
	75–79	0.20	0.16	0.25	10.43	9.93	10.93	0.20	0.16	0.25	10.39	9.89	10.89	0.04	0.00	0.08	0.38
	80–84	0.27	0.22	0.33	7.28	6.78	7.78	0.28	0.22	0.34	7.24	6.74	7.75	0.04	0.00	0.08	0.52
	85 +	0.45	0.39	0.52	4.78	4.25	5.32	0.45	0.39	0.52	4.78	4.25	5.32	0.00	− 0.05	0.05	0.01

**Table 20:** Germany

		Replicated weights						Education-adjusted weights						Bias			
Gender	Age	$\pi$	95% CI		HEX	95% CI		$\pi$	95% CI		HEX	95% CI		∆HEX	95% CI		∆%
Men	50–54	0.55	− 0.13	1.24	21.47	17.78	25.16	0.52	− 0.17	1.21	21.46	17.74	25.19	0.00	− 3.70	3.71	0.02
	55–59	0.18	0.10	0.27	19.76	18.30	21.22	0.18	0.10	0.26	19.58	18.06	21.10	0.18	− 0.03	0.39	0.91
	60–64	0.17	0.10	0.23	16.41	14.94	17.88	0.18	0.10	0.25	16.20	14.66	17.73	0.21	0.04	0.39	1.31
	65–69	0.14	0.08	0.20	13.13	11.60	14.67	0.15	0.08	0.22	12.96	11.36	14.56	0.17	− 0.01	0.35	1.31
	70–74	0.18	0.12	0.24	9.86	8.21	11.51	0.17	0.11	0.23	9.71	7.99	11.44	0.15	− 0.04	0.33	1.49
	75–79	0.22	0.14	0.31	6.90	5.03	8.76	0.22	0.14	0.31	6.66	4.71	8.61	0.24	− 0.02	0.50	3.58
	80–84	0.42	0.29	0.56	4.33	2.00	6.66	0.46	0.32	0.60	4.01	1.58	6.45	0.32	− 0.12	0.75	7.86
	85 +	0.50	0.30	0.71	2.93	− 0.51	6.38	0.55	0.34	0.75	2.68	− 0.94	6.30	0.25	− 0.71	1.21	9.37
Women	50–54	0.13	− 0.01	0.27	26.03	24.86	27.19	0.18	− 0.01	0.37	25.55	24.32	26.78	0.48	0.12	0.83	1.86
	55–59	0.20	0.13	0.28	22.01	21.06	22.96	0.22	0.14	0.30	21.77	20.81	22.73	0.24	0.14	0.35	1.12
	60–64	0.14	0.09	0.19	18.45	17.53	19.37	0.16	0.09	0.23	18.28	17.35	19.21	0.18	0.08	0.27	0.97
	65–69	0.22	0.14	0.29	14.68	13.77	15.60	0.22	0.14	0.29	14.60	13.67	15.52	0.09	− 0.02	0.20	0.59
	70–74	0.22	0.15	0.29	11.36	10.47	12.25	0.25	0.16	0.33	11.28	10.38	12.18	0.08	− 0.02	0.18	0.70
	75–79	0.25	0.16	0.35	8.17	7.27	9.07	0.25	0.15	0.36	8.21	7.31	9.10	− 0.04	− 0.17	0.10	− 0.45
	80–84	0.37	0.22	0.51	5.34	4.44	6.25	0.37	0.21	0.54	5.41	4.50	6.31	− 0.06	− 0.25	0.12	− 1.18
	85 +	0.51	0.37	0.65	3.35	2.46	4.24	0.49	0.34	0.64	3.48	2.59	4.37	− 0.13	− 0.30	0.04	− 3.74

**Table 21:** Hungary

Gender	Age	Replicated weights						Education-adjusted weights						Bias			
		$\pi$	95% CI		HEX	95% CI		$\pi$	95% CI		HEX	95% CI		∆HEX	95% CI		∆%
Men	50–54	0.13	0.05	0.22	18.34	17.48	19.20	0.15	0.05	0.24	18.24	17.37	19.12	0.09	− 0.01	0.20	0.51
	55–59	0.25	0.14	0.37	15.07	14.20	15.94	0.25	0.14	0.37	15.03	14.14	15.91	0.04	− 0.03	0.12	0.29
	60–64	0.20	0.12	0.28	12.67	11.75	13.59	0.21	0.13	0.28	12.64	11.71	13.57	0.03	− 0.04	0.11	0.25
	65–69	0.22	0.11	0.32	10.20	9.17	11.22	0.21	0.12	0.31	10.20	9.16	11.24	0.00	− 0.10	0.10	− 0.02
	70–74	0.18	0.10	0.26	7.84	6.65	9.02	0.19	0.10	0.27	7.83	6.63	9.03	0.01	− 0.13	0.14	0.10
	75–79	0.37	0.18	0.56	5.37	3.89	6.86	0.36	0.18	0.54	5.40	3.90	6.91	− 0.03	− 0.24	0.17	− 0.58
	80–84	0.58	0.37	0.80	3.96	1.88	6.04	0.59	0.36	0.83	3.92	1.81	6.03	0.04	− 0.31	0.38	1.00
	85 +	0.29	0.09	0.49	4.09	0.52	7.65	0.29	0.09	0.49	4.08	0.46	7.70	0.01	− 0.95	0.97	0.17
Women	50–54	0.15	0.05	0.24	23.64	23.01	24.27	0.18	0.05	0.30	23.35	22.71	24.00	0.28	0.22	0.34	1.21
	55–59	0.16	0.08	0.24	19.93	19.33	20.53	0.16	0.09	0.23	19.80	19.20	20.40	0.13	0.09	0.18	0.66
	60–64	0.14	0.05	0.23	16.42	15.83	17.01	0.16	0.06	0.27	16.29	15.70	16.89	0.13	0.09	0.18	0.80
	65–69	0.19	0.06	0.31	12.95	12.36	13.55	0.19	0.08	0.29	12.91	12.32	13.51	0.04	− 0.01	0.09	0.31
	70–74	0.24	0.16	0.32	9.77	9.17	10.36	0.24	0.16	0.32	9.72	9.13	10.32	0.04	− 0.02	0.10	0.43
	75–79	0.26	0.16	0.36	7.03	6.43	7.63	0.27	0.17	0.37	6.95	6.35	7.55	0.09	0.02	0.16	1.23
	80–84	0.48	0.34	0.62	4.58	3.94	5.21	0.48	0.36	0.61	4.54	3.90	5.17	0.04	− 0.05	0.13	0.88
	85 +	0.42	0.25	0.59	3.61	2.88	4.34	0.43	0.26	0.60	3.55	2.82	4.28	0.06	− 0.07	0.18	1.66

**Table 22:** Italy

Gender	Age	Replicated weights						Education-adjusted weights						Bias			
		$\pi$	95% CI		HEX	95% CI		$\pi$	95% CI		HEX	95% CI		∆HEX	95% CI		∆%
Men	50–54	0.01	− 0.01	0.02	26.85	25.95	27.75	0.01	− 0.01	0.03	26.91	26.01	27.81	− 0.06	− 0.17	0.05	− 0.22
	55–59	0.07	0.03	0.10	22.31	21.40	23.22	0.06	0.03	0.10	22.37	21.47	23.28	− 0.06	− 0.15	0.02	− 0.27
	60–64	0.07	0.03	0.10	18.20	17.28	19.13	0.06	0.03	0.09	18.26	17.34	19.18	− 0.06	− 0.13	0.02	− 0.31
	65–69	0.13	0.09	0.17	14.25	13.30	15.21	0.13	0.09	0.17	14.31	13.36	15.26	− 0.06	− 0.14	0.02	− 0.40
	70–74	0.12	0.08	0.16	10.83	9.82	11.84	0.11	0.07	0.15	10.88	9.88	11.89	− 0.05	− 0.14	0.03	− 0.48
	75–79	0.22	0.16	0.28	7.48	6.37	8.60	0.22	0.16	0.27	7.52	6.41	8.63	− 0.04	− 0.15	0.07	− 0.49
	80–84	0.30	0.21	0.38	4.84	3.50	6.19	0.29	0.20	0.38	4.88	3.54	6.22	− 0.03	− 0.20	0.13	− 0.71
	85 +	0.54	0.40	0.67	2.79	0.86	4.72	0.53	0.40	0.67	2.80	0.88	4.72	− 0.01	− 0.37	0.34	− 0.51
Women	50–54	0.09	0.03	0.15	28.76	28.05	29.48	0.08	0.03	0.14	28.84	28.12	29.55	− 0.07	− 0.14	0.00	− 0.25
	55–59	0.09	0.05	0.12	24.47	23.77	25.17	0.08	0.05	0.12	24.52	23.82	25.22	− 0.05	− 0.11	0.01	− 0.19
	60–64	0.09	0.06	0.12	20.24	19.55	20.93	0.09	0.06	0.12	20.29	19.60	20.98	− 0.05	− 0.10	0.00	− 0.23
	65–69	0.10	0.07	0.14	16.12	15.42	16.81	0.10	0.07	0.14	16.16	15.47	16.86	− 0.05	− 0.10	0.01	− 0.29
	70–74	0.16	0.12	0.21	12.16	11.46	12.85	0.16	0.12	0.21	12.21	11.51	12.90	− 0.05	− 0.11	0.01	− 0.41
	75–79	0.26	0.19	0.32	8.60	7.89	9.31	0.25	0.18	0.31	8.66	7.95	9.37	− 0.05	− 0.12	0.02	− 0.61
	80–84	0.36	0.27	0.45	5.71	4.98	6.45	0.36	0.27	0.45	5.73	5.00	6.46	− 0.02	− 0.11	0.08	− 0.27
	85 +	0.52	0.40	0.63	3.62	2.82	4.41	0.52	0.40	0.64	3.61	2.81	4.40	0.01	− 0.11	0.13	0.24

**Table 23:** Poland

Gender	Age	Replicated weights						Education-adjusted weights						Bias			
		$\pi$	95% CI		HEX	95% CI		$\pi$	95% CI		HEX	95% CI		∆HEX	95% CI		∆%
Men	50–54	0.00	–	–	21.12	19.95	22.28	0.00	–	–	21.37	20.20	22.53	− 0.25	− 1.41	0.92	− 1.17
	55–59	0.18	0.11	0.24	17.04	15.82	18.26	0.16	0.10	0.23	17.30	16.08	18.53	− 0.26	− 0.39	− 0.13	− 1.51
	60–64	0.18	0.13	0.24	14.06	12.78	15.34	0.18	0.12	0.24	14.27	12.98	15.55	− 0.21	− 0.35	− 0.08	− 1.47
	65–69	0.19	0.12	0.26	11.30	9.91	12.69	0.17	0.10	0.24	11.50	10.10	12.90	− 0.20	− 0.36	− 0.03	− 1.73
	70–74	0.24	0.15	0.33	8.74	7.17	10.32	0.24	0.15	0.32	8.88	7.29	10.47	− 0.14	− 0.37	0.09	− 1.57
	75–79	0.30	0.19	0.40	6.56	4.67	8.44	0.30	0.17	0.43	6.69	4.78	8.59	− 0.13	− 0.43	0.17	− 1.92
	80–84	0.39	0.25	0.53	4.82	2.30	7.34	0.35	0.20	0.49	5.04	2.49	7.58	− 0.21	− 0.72	0.29	− 4.25
	85 +	0.31	0.15	0.47	3.92	− 0.14	7.98	0.30	0.14	0.46	3.98	− 0.12	8.08	− 0.06	− 1.08	0.96	− 1.58
Women	50–54	0.02	− 0.02	0.07	24.11	23.19	25.02	0.04	− 0.03	0.10	24.16	23.23	25.08	− 0.05	− 0.25	0.15	− 0.21
	55–59	0.13	0.09	0.18	19.62	18.72	20.53	0.13	0.08	0.18	19.73	18.83	20.63	− 0.11	− 0.19	− 0.02	− 0.55
	60–64	0.11	0.06	0.15	15.78	14.88	16.68	0.10	0.06	0.14	15.89	14.99	16.79	− 0.11	− 0.20	− 0.03	− 0.72
	65–69	0.21	0.14	0.28	11.90	10.98	12.82	0.20	0.13	0.28	11.98	11.06	12.90	− 0.08	− 0.19	0.03	− 0.67
	70–74	0.38	0.29	0.48	8.60	7.68	9.51	0.39	0.30	0.49	8.65	7.73	9.57	− 0.05	− 0.17	0.06	− 0.63
	75–79	0.40	0.29	0.52	6.23	5.33	7.14	0.40	0.28	0.51	6.33	5.43	7.24	− 0.10	− 0.24	0.04	− 1.56
	80–84	0.49	0.38	0.60	4.14	3.23	5.04	0.47	0.35	0.59	4.21	3.30	5.11	− 0.07	− 0.21	0.07	− 1.70
	85 +	0.61	0.45	0.77	2.69	1.61	3.77	0.61	0.45	0.77	2.66	1.58	3.74	0.03	− 0.22	0.28	0.97

**Table 24:** Portugal

Gender	Age	Replicated weights						Education-adjusted weights						Bias			
		$\pi$	95% CI		HEX	95% CI		$\pi$	95% CI		HEX	95% CI		∆HEX	95% CI		∆%
Men	50–54	0.05	0.00	0.10	25.92	24.74	27.09	0.05	0.00	0.09	25.92	24.75	27.08	0.00	− 0.17	0.17	0.01
	55–59	0.18	0.03	0.33	21.91	20.72	23.11	0.18	0.02	0.35	21.89	20.71	23.07	0.02	− 0.11	0.15	0.09
	60–64	0.04	0.01	0.07	18.67	17.46	19.87	0.04	0.01	0.08	18.66	17.46	19.85	0.01	− 0.12	0.14	0.05
	65–69	0.22	0.07	0.36	14.82	13.55	16.09	0.23	0.07	0.39	14.84	13.59	16.09	− 0.02	− 0.15	0.12	− 0.12
	70–74	0.09	0.03	0.15	12.05	10.71	13.40	0.08	0.02	0.14	12.14	10.82	13.47	− 0.09	− 0.26	0.08	− 0.75
	75–79	0.22	0.10	0.35	8.92	7.40	10.43	0.22	0.09	0.34	8.97	7.47	10.47	− 0.05	− 0.28	0.18	− 0.56
	80–84	0.24	0.05	0.44	6.84	4.99	8.69	0.23	0.02	0.45	6.85	5.03	8.68	− 0.01	− 0.39	0.36	− 0.20
	85 +	0.03	− 0.02	0.09	5.66	2.94	8.39	0.05	− 0.02	0.11	5.60	2.91	8.29	0.06	− 0.79	0.92	1.11
Women	50–54	0.21	0.07	0.34	26.16	25.22	27.10	0.21	0.07	0.36	27.11	26.18	28.03	**− **0.95	**− **1.05	**− **0.85	**− **3.50
	55–59	0.09	0.03	0.14	22.47	21.57	23.38	0.09	0.03	0.14	23.47	22.58	24.35	**− **0.99	**− **1.08	**− **0.90	**− **4.23
	60–64	0.16	0.05	0.26	18.26	17.36	19.16	0.10	0.04	0.16	19.26	18.38	20.14	**− **1.01	**− **1.10	**− **0.91	**− **5.22
	65–69	0.10	0.05	0.15	14.45	13.56	15.33	0.10	0.04	0.16	15.20	14.33	16.08	**− **0.76	**− **0.84	**− **0.67	**− **4.97
	70–74	0.27	0.10	0.44	10.41	9.52	11.30	0.20	0.08	0.32	11.20	10.31	12.08	**− **0.78	**− **0.90	**− **0.67	**− **7.01
	75–79	0.21	0.08	0.34	7.37	6.51	8.22	0.19	0.07	0.31	7.83	6.96	8.69	**− **0.46	**− **0.58	**− **0.34	**− **5.88
	80–84	0.41	0.23	0.59	4.17	3.29	5.04	0.38	0.19	0.56	4.57	3.67	5.47	**− **0.40	**− **0.55	**− **0.26	**− **8.83
	85 +	0.72	0.50	0.93	2.02	1.07	2.98	0.67	0.38	0.96	2.38	1.38	3.37	**− **0.35	**− **0.56	**− **0.14	**− **14.83

**Table 25:** Slovenia

Gender	Age	Replicated weights						Education-adjusted weights						Bias			
		$\pi$	95% CI		HEX	95% CI		$\pi$	95% CI		HEX	95% CI		∆HEX	95% CI		∆%
Men	50–54	0.07	0.03	0.11	26.01	25.15	26.87	0.07	0.03	0.11	25.91	25.04	26.78	0.10	0.00	0.20	0.38
	55–59	0.05	0.03	0.08	21.98	21.12	22.84	0.06	0.03	0.08	21.90	21.02	22.77	0.08	0.01	0.16	0.38
	60–64	0.09	0.04	0.13	18.19	17.30	19.08	0.09	0.04	0.13	18.11	17.20	19.01	0.08	− 0.01	0.17	0.45
	65–69	0.08	0.04	0.12	14.72	13.79	15.66	0.09	0.04	0.13	14.64	13.69	15.59	0.08	− 0.02	0.18	0.56
	70–74	0.17	0.11	0.24	11.46	10.45	12.47	0.18	0.11	0.25	11.38	10.35	12.40	0.08	− 0.04	0.19	0.68
	75–79	0.20	0.12	0.28	8.95	7.80	10.09	0.20	0.13	0.28	8.89	7.73	10.04	0.06	− 0.09	0.21	0.67
	80–84	0.14	0.06	0.21	7.04	5.61	8.48	0.15	0.06	0.23	6.99	5.54	8.45	0.05	− 0.19	0.29	0.71
	85 +	0.04	− 0.01	0.09	5.54	3.34	7.74	0.04	− 0.01	0.09	5.54	3.30	7.77	0.00	− 0.52	0.52	0.03
Women	50–54	0.12	0.07	0.16	29.09	28.40	29.78	0.13	0.08	0.18	28.84	28.14	29.53	0.25	0.19	0.32	0.88
	55–59	0.13	0.03	0.23	25.04	24.37	25.70	0.15	0.03	0.26	24.84	24.17	25.51	0.19	0.14	0.25	0.78
	60**–**64	0.10	0.04	0.16	21.15	20.50	21.80	0.11	0.04	0.18	21.03	20.37	21.68	0.12	0.07	0.18	0.59
	65–69	0.16	0.07	0.25	17.24	16.60	17.88	0.17	0.08	0.26	17.15	16.51	17.79	0.09	0.02	0.15	0.52
	70–74	0.17	0.07	0.28	13.78	13.16	14.39	0.18	0.07	0.29	13.72	13.10	14.34	0.05	− 0.01	0.11	0.38
	75–79	0.22	0.11	0.32	10.48	9.88	11.09	0.21	0.11	0.32	10.48	9.87	11.08	0.01	− 0.06	0.08	0.08
	80–84	0.17	0.10	0.23	7.89	7.29	8.48	0.17	0.09	0.24	7.87	7.27	8.47	0.01	− 0.06	0.09	0.16
	85 +	0.21	0.10	0.32	5.56	4.86	6.26	0.21	0.10	0.32	5.55	4.85	6.26	0.01	− 0.12	0.13	0.17

**Table 26:** Spain

Gender	Age	Replicated weights						Education-adjusted weights						Bias			
		$\pi$	95% CI		HEX	95% CI		$\pi$	95% CI		HEX	95% CI		∆HEX	95% CI		∆%
Men	50–54	0.03	0.00	0.06	29.05	28.47	29.63	0.03	0.00	0.05	29.09	28.51	29.67	− 0.04	− 0.11	0.02	− 0.14
	55–59	0.05	0.02	0.08	24.79	24.21	25.37	0.04	0.01	0.07	24.82	24.24	25.40	− 0.02	− 0.08	0.03	− 0.10
	60–64	0.05	0.02	0.08	20.81	20.23	21.40	0.05	0.02	0.07	20.82	20.23	21.40	0.00	− 0.06	0.05	− 0.02
	65–69	0.04	0.01	0.07	17.04	16.44	17.64	0.04	0.01	0.07	17.02	16.42	17.62	0.02	− 0.03	0.07	0.12
	70–74	0.04	0.01	0.06	13.40	12.77	14.04	0.03	0.01	0.05	13.39	12.76	14.03	0.01	− 0.05	0.07	0.08
	75–79	0.10	0.06	0.14	10.01	9.31	10.72	0.11	0.07	0.14	9.98	9.27	10.69	0.03	− 0.03	0.09	0.30
	80–84	0.16	0.10	0.22	7.24	6.40	8.08	0.16	0.10	0.22	7.24	6.39	8.08	0.01	− 0.09	0.10	0.08
	85 +	0.19	0.11	0.26	5.30	4.14	6.47	0.19	0.11	0.26	5.30	4.14	6.47	0.00	− 0.15	0.15	0.01
Women	50–54	0.01	0.00	0.02	34.02	33.58	34.45	0.01	0.00	0.03	34.01	33.58	34.45	0.00	− 0.04	0.04	0.01
	55–59	0.01	0.00	0.03	29.40	28.97	29.84	0.01	0.00	0.03	29.41	28.97	29.84	0.00	− 0.04	0.03	− 0.01
	60–64	0.01	0.00	0.02	24.86	24.43	25.30	0.01	0.00	0.02	24.86	24.43	25.30	0.00	− 0.04	0.04	0.00
	65–69	0.04	0.02	0.06	20.36	19.92	20.80	0.04	0.02	0.06	20.36	19.92	20.80	0.00	− 0.04	0.04	0.01
	70–74	0.05	0.02	0.08	16.13	15.69	16.56	0.05	0.02	0.08	16.12	15.68	16.56	0.01	− 0.03	0.05	0.05
	75–79	0.09	0.05	0.12	12.13	11.69	12.57	0.09	0.06	0.13	12.12	11.68	12.56	0.00	− 0.03	0.04	0.02
	80–84	0.14	0.09	0.19	8.65	8.19	9.10	0.14	0.09	0.19	8.66	8.20	9.11	− 0.01	− 0.06	0.04	− 0.10
	85 +	0.25	0.19	0.32	5.93	5.43	6.43	0.25	0.18	0.32	5.95	5.46	6.45	− 0.02	− 0.08	0.03	− 0.39
